# Early and Sensitive Detection of Cisplatin-Induced Kidney Injury Using Novel Biomarkers

**DOI:** 10.1016/j.ekir.2025.01.035

**Published:** 2025-02-05

**Authors:** Michael Strader, Gary Friedman, Xavier Benain, Nunzio Camerlingo, Stefan Sultana, Shiran Shapira, Nadir Aber, Patrick T. Murray

**Affiliations:** 1 Department of Medicine, School of Medicine, University College Dublin, Dublin, Ireland; 2 Global Biometrics and Data Management, Pfizer, Inc., Cambridge, Massachusetts, USA; 3 Biostastics and Programming, Sanofi S.A., Paris, France; 4 Patient Safety Center of Excellence, AstraZeneca PLC, Cambridge, UK; 5 Health Promotion Center and Integrated Cancer Prevention Center, Tel Aviv Sourasky Medical Center and Tel Aviv University, Tel Aviv, Israel

**Keywords:** biomarkers, drug-induced kidney injury, subclinical acute kidney injury

## Abstract

**Introduction:**

We evaluated a panel of novel urinary and serum biomarkers (BMs) for early and sensitive detection of cisplatin drug-induced kidney injury (DIKI) in patients with cancer, comparing their diagnostic accuracy with standard BMs (SBMs).

**Methods:**

In this prospective exploratory observational study, 105 patients treated with cisplatin (“treated” with > 65 mg/m^2^/cycle), 20 non-cisplatin treated cancer controls (“nontreated”), and 34 “healthy” controls were enrolled. The treated group’s serum and urine samples were collected predose, after 12 hours, and on days 1, 2, 4, 7, 14, and 21. SBMs and novel BMs (NBMs; 8 urinary, 1 serum) were measured, comparing accuracy, percent changes from baseline (PCFBs), and median time to peak values between treated patients and nontreated cancer controls. Blinded adjudication of the treated group’s BM profiles occurred at 2 stages for DIKI diagnosis.

**Results:**

All urinary NBMs had significant PCFBs in the treated group compared with the nontreated cancer control group; most accurately detected cisplatin exposure (area under the receiver operating characteristics [ROC] curve [AUROC] > 0.8). NBMs peaked earlier. In stage 1 adjudication (SBMs) of the treated group, PCFB of urinary NBMs showed no difference between DIKI (*n* = 24) and no-DIKI (*n* = 71) groups except for neutrophil gelatinase-associated lipocalin (NGAL) and cystatin C (CYSC). In treated participants, all BMs showed greater PCFBs than control groups, regardless of stage 1 DIKI adjudication. Stage 2 (SBMs and NBMs), DIKI incidence (*n* = 63) increased by 41%, with most BMs having an AUROC > 0.80 compared with the nontreated cancer control group.

**Conclusion:**

NBMs accurately and timely detected cisplatin exposure and identified “sub-clinical” DIKI undetected by standard acute kidney injury (AKI) criteria, highlighting the limitations of current functional BMs in estimating the true DIKI incidence.

## Graphical abstract

**Figure:**
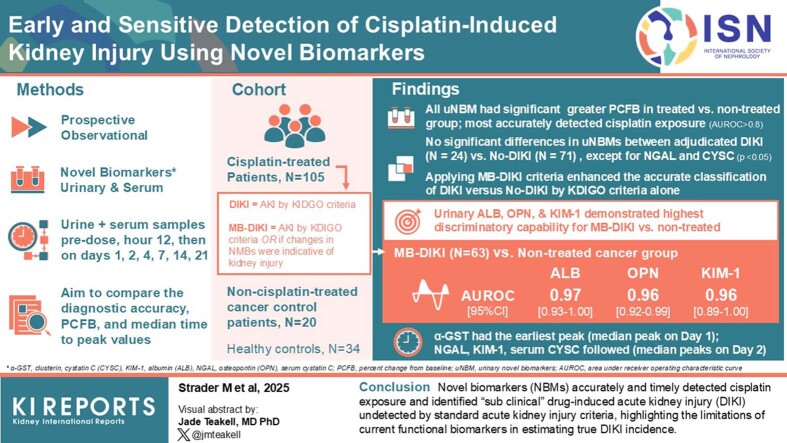

DIKI, a subphenotype of AKI, is characterized by the involvement of 1 or more nephrotoxic drugs in its pathogenesis.^1^ It is estimated that within the hospital, 19% to 26% of all kidney injuries are due to nephrotoxicity.^2^ The mechanism by which DIKI occurs is broadly categorized into systemic or kidney hemodynamics and tubular or structural injury.^3^ Cisplatin, a platinum-derived alkylating chemotherapy agent commonly used for the treatment of solid tumors, is known to cause a dose-dependent DIKI in approximately 25% to 30% of the population.^4^, ^5^, ^6^

The development of AKI can trigger maladaptive responses that result in significant patient morbidity and mortality outcomes, including the development of chronic kidney disease (CKD), cardiovascular disease, and the requirement for acute and sometimes chronic dialysis.^1^^,^^7^, ^8^, ^9^ In patients with cancer, the emergence of DIKI or CKD can lead to medication discontinuation, dose adjustments, and limitations in life-saving treatments based on residual kidney function.^5^ Moreover, DIKI often manifests as a late-stage factor, leading to the discontinuation of new drugs in the development process.^10^^,^^11^

Currently, the diagnosis and staging of AKI is based on the Kidney Disease: Improving Global Outcomes (KDIGO) criteria, which include the current standard clinical BMs to estimate kidney function: serum creatinine (SCr) and urine output.^12^ However, both functional BMs have limitations, including delayed measurable changes from the time of injury, an inability to identify the site of kidney injury in the nephron, and failure to differentiate between purely functional reversible “prerenal” AKI caused by kidney hypoperfusion and the presence of kidney dysfunction with underlying acute kidney tubular damage.^1^^,^^3^

To overcome current standard functional BM limitations, the 23^rd^ Acute Disease Qualitative Initiative meeting proposed augmenting the KDIGO AKI criteria with NBMs.^3^^,^^13^ This inclusion not only aims to address the often overlooked “subclinical” AKI group, but also lays the groundwork for a novel framework proposed at the 23rd Acute Disease Qualitative Initiative meeting.^1^^,^^3^ This novel framework (2 × 2 table) classifies AKI into 4 categories based on functional and damage BMs status, considering both positivity and negativity for each type of BM.^3^^,^^13^^,^^14^

Increasingly, NBMs such as kidney injury molecule-1 (KIM-1), NGAL, and CYSC, as well as metabolomic or proteomic profiles, are being utilized in drug development and clinical practice to detect AKI earlier and have greater sensitivity than conventional clinical measures of kidney function.^6^^,^^15^^,^^16^

This exploratory observational study, conducted within the framework of the Innovative Medicines Initiative (IMI)/Safer and Faster Evidence-Based Translation (SAFE-T) project, aimed to evaluate the potential of a panel of NBMs for the early and sensitive detection of nephrotoxin exposure and DIKI in a cancer population. We aimed to compare the performance of these NBMs with SBMs. In addition, the study explored the sensitivity of the NBMs and their PCFBs in patients diagnosed with cisplatin-induced DIKI based on adjudication using SBMs alone and SBMs with NBMs.

## Methods

This prospective exploratory, nonrandomized, longitudinal cohort study included patients from 3 different cohorts that participated in the IMI/SAFE-T project. The study involved patients from clinical sites including the departments of Nephrology and Oncology at Charité Hospital, Berlin, and the Integrated Cancer Prevention Center and Department of Oncology at the Tel Aviv Sourasky Medical Centre, Tel Aviv, Israel.

The IMI/SAFE-T consortium collected samples and generated BM data from 2009 to 2015 with the aim of developing tools for predicting, detecting, and monitoring DIKI (Supplementary Material).^17^ Our request for participant-level data were granted by TranSMART and fulfilled by Information Technology for Translational Medicine. The project was carried out in accordance with The Code of Ethics of the World Medical Association (Declaration of Helsinki).

### Inclusion Criteria

#### All Groups

For all groups, patients aged ≥ 18 years were included.

#### Cisplatin Treated Group (“Treated” Group)

This group included patients with documented cancer diagnosis (Supplementary Table S1) and normal kidney function (estimated glomerular filtration rate [eGFR]> 60 ml/min per 1.73 m^2^) receiving their first high-dose cisplatin therapy (> 65 mg/m^2^/cycle) as part of their standard treatment protocol.

#### Non-Cisplatin Treated Cancer Control Group (“Nontreated” Group):

These were patients with documented cancer diagnosis (Supplementary Table S1) who received non-cisplatin treatment modalities (Supplementary Table S2) for cancer.

#### Healthy Control Group (“Healthy” Group)

These were healthy patients with no active diseases.

### Exclusion Criteria

#### All Groups

One exclusion criterion was CKD, which was defined by microalbuminuria (> 30 μg/mg urinary creatinine) or eGFR < 60 ml/min per 1.73 m^2^. The eGFR was calculated using the 2009 creatinine-based CKD-Epidemiology Collaboration formula, which was accepted standard at the time of patient recruitment.

Another exclusion criterion was the regular coadministration of creatine supplements, drugs that alter the tubular secretion of creatinine (e.g., trimethoprim, cimetidine), or nonsteroidal antiinflammatory medications (e.g. ibuprofen, diclofenac, naproxen) within 7 days before screening until the last sample collection timepoint.

Lastly, patients who had a major surgery from 1 month before screening until the last sample collection timepoint were excluded.

### Serum and Urine Sample Collection

For all groups, at each timepoint, 10 ml of serum and 40 ml of urine were collected.

In the treated group, serum and urine samples were collected at the following time points: predose, within 12 hours of cisplatin dose, and on days 1, 2, 4 (+/− 1), 7 (+/−1), 14 (+/− 3), and 21 (+/− 7) after cisplatin dose. In the nontreated cancer control group, serum and urine samples were collected within 3 weeks of the initial screening. For this group, samples were obtained on their first visit (day 0) and 2 days later (± 1 day).

Healthy patients were recruited from 2 prospective studies. In one study, participants attended 3 separate visits, 1 week apart, during which serum and urine samples were collected. In the other study, serum and urine samples were collected from healthy patients during 3 visits over a 2- to 4-week period.

Urine samples were immediately centrifuged at 2000 g for 10 minutes. Supernatants and serum samples were aliquoted and frozen at −70 °C. Samples were shipped on dry ice to a central storage facility and were maintained at −70 °C until processing.

### BM Selection

Eight novel candidate urinary BMs and 1 novel serum BM were selected using the following steps in a preliminary stage gate analysis of 22 BMs (Supplementary Tables S3 and S4) and 2 assays (Supplementary Tables S5 and S6): (i) BMs were ranked based on ROC curve performance values (AUROC for change from baseline), (ii) higher weighting was given for BMs that were qualified for preclinical use, (iii) represent different pathophysiological processes (i.e., tubular reabsorption, necrosis proteins, inflammatory mediators, and tissue repair markers), and (iv) baseline BM levels were similar between the treated group and the healthy group.

BMs were normalized to urinary creatinine to minimize variability across sample collection timepoints.^18^, ^19^, ^20^

### Assays

Urine albumin (ALB), urine total protein, blood urea nitrogen, serum CYSC, and SCr levels were measured using well-established routine laboratory tests on clinical analyzers.

Before stage gate analysis, SAFE-T laboratories with assay development capabilities assessed and validated up to 2 assays for each NBM of interest. A generic SAFE-T standard validation procedure, based on the fit-for-purpose concept, was developed to evaluate technical performance against the predefined purpose.^21^^,^^22^ The stringency of performance verification varied depending on the intended use of the assay. Validation procedures adhered to guidelines issued by regulatory authorities, including the European Medicines Agency (2009), the US Food and Drug Administration (2013), and the Clinical and Laboratory Standards Institute. Limit of detection, limit of quantification, intra- or inter-assay precision, parallelism and/or dilutional linearity, analyte stability, assay dynamic range, and spike-in recovery were assessed during assay validation.

The assays included single assay enzyme-linked immunosorbent assay and Multiplex (Luminex) (Supplementary Tables S5 and S6). For BMs with 2 assay methods, we compared the assay methods using scatter plots of paired individual BM values, and the assay with the best performance was selected for stage gate analysis.

Quality control was applied during the sample screening procedure to ensure data reliability and comparability throughout the SAFE-T/IMI project.

### Adjudication

Three expert nephrologists independently reviewed deidentified datasets at 2 stages. At stage 1, they assessed serial measurements of SCr, eGFR, and their PCFBs to adjudicate the presence or absence of DIKI (AKI or no-AKI) using KDIGO AKI criteria. Responses were restricted to the following: AKI (“DIKI”), no-AKI (“No-DIKI”), or uncertain. Consensus was declared when 2 of 3 of the responses concurred.

In stage 2, a similar adjudication process was repeated, but with deidentified datasets containing PCFBs for both standard and NBMs and using modified KDIGO AKI criteria (standard creatinine-staging criteria with NBMs raw values and PCFBs). The adjudicators categorized patients as meeting AKI criteria if they satisfied the SCr thresholds or if changes in NBMs were indicative of kidney injury. Responses were restricted to the following: AKI (“multiple biomarker-DIKI” [MB-DIKI]), no-AKI (“no-MB-DIKI”), or uncertain.

### Statistical Analysis

Demographic data such as patient age, biological sex, body mass index, cancer types, and diabetes status were collected at time of inclusion. At the time of analysis, eGFR was calculated using the creatinine-based CKD-Epidemiology Collaboration 2021 formula. Data from all the patients were included in analysis, whether they prematurely withdrew or not. No scheduled follow-up occurred for the patients outside of their standard-of-care provided by their institution.

Sample size estimation was performed using simulations comparing the AUROC of single BMs versus SCr, applying the binormal Non–Gold Standard Bayesian method.^23^ The calculation assumed a beta (30, 70) prior distribution for DIKI incidence in the treated group, a 0.5 correlation between the test and reference BMs, and a 2-sided type-1 error rate of 5%. The planned sample size included 100 cisplatin-treated patients, 20 nontreated patients, and 30 healthy controls, determined to provide sufficient power for comparing the discriminatory performance of NBMs with the reference biomarker. CIs were used to control type-1 error and ensure reliable interpretation of results.

We hypothesized that NBMs would increase earlier and more sensitively in patients developing DIKI compared with SCr, including those with DIKI defined by the KDIGO AKI criteria or modified KDIGO AKI criteria. We conducted 2 primary analyses to test this hypothesis. Our first primary analysis compared the change from baseline in established functional BMs and NBMs between the treated group and the nontreated cancer control group to determine whether the NBMs could sensitively differentiate cisplatin nephrotoxin exposure from non-cisplatin exposure in a cancer population. Our second primary analysis, which aimed to investigate the NBM changes in a clinical AKI and no-AKI population, compared the PCFBs of NBMs in cases of DIKI, defined by blinded expert adjudication at the 2 stages, to the adjudicated no-DIKI group and 2 control groups.

Urinary BMs were corrected using urinary creatinine values (indexed to urine creatinine to correct for variability of urinary tonicity). The primary endpoint was the PCFBs in postbaseline maximum values for urinary creatinine-corrected BMs (minimum values for eGFR). PCFB was used to account for baseline differences and control for any time component confounder. For each BM, empirical ROC curves were generated to estimate AUROCs and their 95% confidence intervals (CIs), comparing treated versus nontreated controls and adjudicated MB-DIKI versus nontreated controls. The best thresholds (Youden index) were identified, along with their sensitivity and specificity. All ROC curves, estimates, and CIs were obtained using bootstrap.

Group comparisons were performed using nonparametric tests. The Kruskal-Wallis test was used to assess differences in BM concentrations across groups. When significant differences were identified (*P* < 0.1), pairwise comparisons were conducted using *post hoc* Dunn's tests with Bonferroni correction for multiple comparisons. A *P*-value < 0.05 was considered statistically significant. BM concentrations were visualized using boxplots, with medians, interquartile ranges, whiskers, and outliers clearly represented. BM kinetics were assessed using time-profile plots of median values and interquartile ranges for treated patients. All statistics were calculated using SAS software^24^ 9.4 and R statistical software^25^ (v. 3.4.2).

## Results

### Characteristics of Unadjudicated, Stage 1 Adjudicated, and Control Groups

The treated group consisted of 105 patients receiving their first dose of i.v. cisplatin (mean: 139.56, SD: 62.09 mg/m^2^). The control groups included 20 nontreated patients and 34 healthy controls. The treated group primarily comprised patients with lung cancer (*n* = 50 [60.24%]), hematological cancer (*n* = 13 [15.66%]), and oropharyngeal cancer (*n* = 12 [14.46%]). In contrast, the nontreated group primarily included patients with neuroendocrine tumors (*n* = 9 [45%]), liver cancers (*n* = 5 [25%]), and gastrointestinal cancers (*n* = 5 [25%]) (Supplementary Table S1).

In Table 1, we outline the demographic and patient characteristics at baseline. The treated group is presented in its unadjudicated and stage 1 adjudicated forms to demonstrate the characteristics of the DIKI and no-DIKI group.

**Table 1:** Demographics and patient characteristics at baseline

Characteristics	Treated group			Control groups	
	Treated-All (*N* = 105)	Stage 1 adjudicated DIKI (*n* = 24)	Stage 1 adjudicated no-DIKI (*n* = 71)	Nontreated group (*n* = 20)	Healthy group (*n* = 34)
Age (yrs)					
Mean (SD)	59.5 (10.6)	61.54 (9.17)	58.21 (11.43)	63.4 (10.7)	49.5 (16.5)
Sex, *n* (%)					
Female	34 (32.4)	6 (25%)	24 (33.8)	9 (45.0)	17 (50)
Race, *n* (%)					
Caucasian/White	100 (95.2)	24 (100)	67 (94.4)	19 (95.0)	29 (85.3)
Black	1 (1.0)	-	1 (1.4)	-	-
Asian	1 (1.0)	-	-	-	1 (2.9)
Other	3 (2.8)	-	3 (4.2)	1 (5.0)	4 (11.8)
BMI (kg/m^2^)					
Mean (SD)	25.37 (4.50)	25.29 (4.16)	24.8 (4.11)	26.14 (4.76)	23.7 (2.70)
Diabetes mellitus					
[*n* (%)]	12 (11.4)	6 (25)	5 (7.04)	6 (30)	0 (0)
Baseline kidney markers					
Serum creatinine (mg/dl) mean (SD)	0.83 (0.24)	0.78 (0.23)	0.83 (0.25)	0.91 (0.35)	0.86 (0.14)
eGFR (ml/min per 1.73 m^2^) mean (SD)	101.07 (29.35)	106.73 (24.47)	100.53 (31.86)	79.83 (22.93)	101.07 (29.35)
Blood urea nitrogen (mmol/l) mean (SD)	23.67 (10.17)	28.38 (11.07)	22.25 (9.70)	36.35 (22.25)	30.12 (9.09)

BMI, body mass index; DIKI, drug-induced kidney injury; eGFR: estimated glomerular filtration rate.

Treated group includes uncertain patients during adjudication patients and insufficient BM data.

The DIKI and no-DIKI group were similar for age, sex, body mass index, and eGFR. Between the treated and nontreated cancer control groups, age and body mass index were similar. On an average, the healthy control group was younger (49.5, SD: 16.5 years) and had lower body mass index (23.7 kg/m^2^, SD: 2.70) compared with all other groups. The treated cancer group composed of fewer females (31.6%) compared with the nontreated cancer control group (45%) and healthy control group (50%). Race was similar across all groups, with the majority being White participants.

At baseline, SCr was significantly higher (*P* = 0.003) in the nontreated cancer control group (mean: 0.91, SD 0.35 mg/dl) than in the treated (mean 0.83, SD: 0.24) and healthy control groups (mean: 0.86, SD: 0.14). In addition, eGFR was significantly lower (*P* = 0.026) in the non treated cancer control group (mean 79.83, SD: 22.93 ml/min per 1.73 m^2^) than in the treated (mean: 101.07, SD: 29.35 ml/min per 1.73 m^2^) and healthy control groups (mean 101.07, SD: 29.35 ml/min per 1.73 m^2^). Blood urea nitrogen levels were higher in the nontreated cancer control group (mean: 36.35, SD: 22.25 mmol/l) than in the treated group (mean 23.67, SD: 10.17 mmol/l) and healthy control group (30.12, SD: 9.09 mmol/l), but the difference was not statistically significant. The observed trends in demographic data remained consistent even after stratifying the treated group at stage 1 into the adjudicated DIKI and no-DIKI subgroups (Table 1).

### Performance of BMs in Detecting Cisplatin Exposure

**Figure 1:**
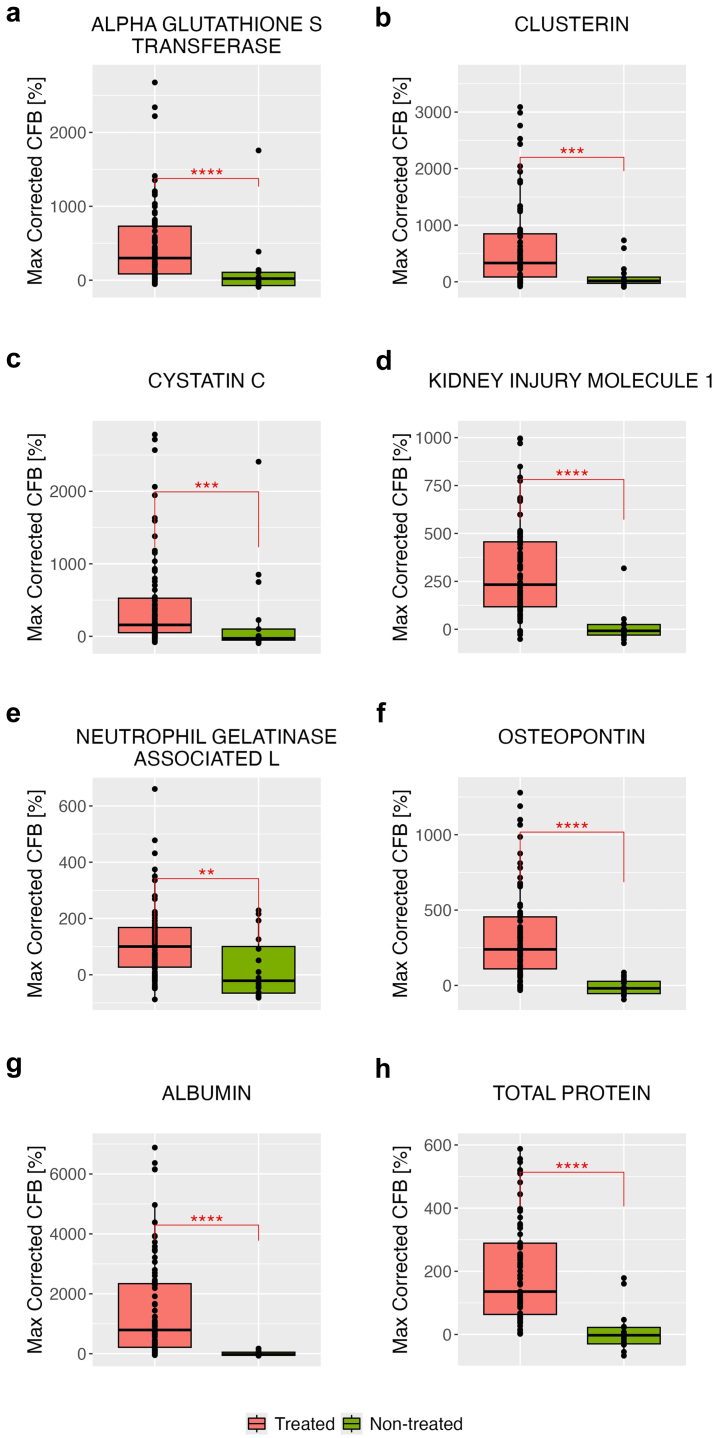
Box-and-whisker plots of urinary biomarkers percent change from baseline in treated and non-treated cancer control groups. The box-and-whisker plots depict the 25th and 75th interquartile ranges, median, and total observed ranges. Statistical significance between groups is indicated by *P*-values obtained from the Kruskal-Wallis test. Symbols represent significance levels: ∗*P* < 0.05, ∗∗*P* < 0.01, ∗∗∗*P* < 0.001, ∗∗∗∗*P* < 0.0001. There was a significant change from baseline in all urinary biomarkers compared to non-treated cancer control patients. (a) Alpha glutathione S transferase, (b) clusterin, (c) cystatin C, (d) kidney injury molecule-1 (KIM-1), (e) neutrophil gelatinase-associated lipocalin (NGAL), (f) osteopontin, (g) albumin, and (h) total protein. CFB, change from baseline.

To evaluate the ability of the NBMs to detect cisplatin exposure in patients with cancer, one of the core objectives of the SAFE-T/IMI project, we analyzed the maximum PCFB in BM values (Figure 1). Notably, most BMs demonstrated good performance, with AUROC values exceeding 0.8. Among these, KIM-1, urinary ALB, and urinary osteopontin (OPN) were the most promising with AUROC of 0.95 (Table 2). Furthermore, all BMs in the treated group showed a significant change from baseline compared with the nontreated cancer control group (Figure 1).

**Table 2:** Urinary and serum biomarker Levels and Diagnostic Accuracy in Treated vs. Non-Treated cancer control group

Biomarker	Treated group	Nontreated group	AUROC and 95% CIs
Osteopontin	88	17	0.95 [0.90–0.98]
Albumin	92	17	0.95 [0.90–0.98]
KIM-1	88	17	0.95 [0.87–0.99]
Total protein	95	17	0.93 [0.84–0.99]
α-GST	88	17	0.84 [0.71–0.93]
Cystatin C	88	17	0.79 [0.64–0.90]
Clusterin	88	17	0.78 [0.60–0.90]
NGAL	88	17	0.71 [0.52–0.85]
Serum cystatin C	103	16	0.92 [0.85–0.97]
Serum creatinine	103	17	0.84 [0.74–0.92]
eGFR	103	17	0.84 [0.73–0.92]

AUROC, area under the receiver operating characteristics curve; CI, confidence interval; eGFR, estimated glomerular filtration rate; KIM-1, kidney injury molecule-1; NGAL, neutrophil gelatinase-associated lipocalin; α-GST, α- glutathione s-transferase.

Missing data were due to incomplete data sets. Urinary biomarkers were creatinine-corrected. albumin (mg/mmol), clusterin (μg/mmol), eGFR (ml/min per 1.73 m^2^), KIM-1 (μg/mmol), NGAL (ng/mmol), osteopontin (μg/mmol), serum creatinine (mg/dl), serum cystatin c (mg/l), total protein (mg/mmol), urine cystatin c (μg/mmol), and α-GST(μg/mmol).

### Time to Peak of NBMs post cisplatin exposure

**Figure 2:**
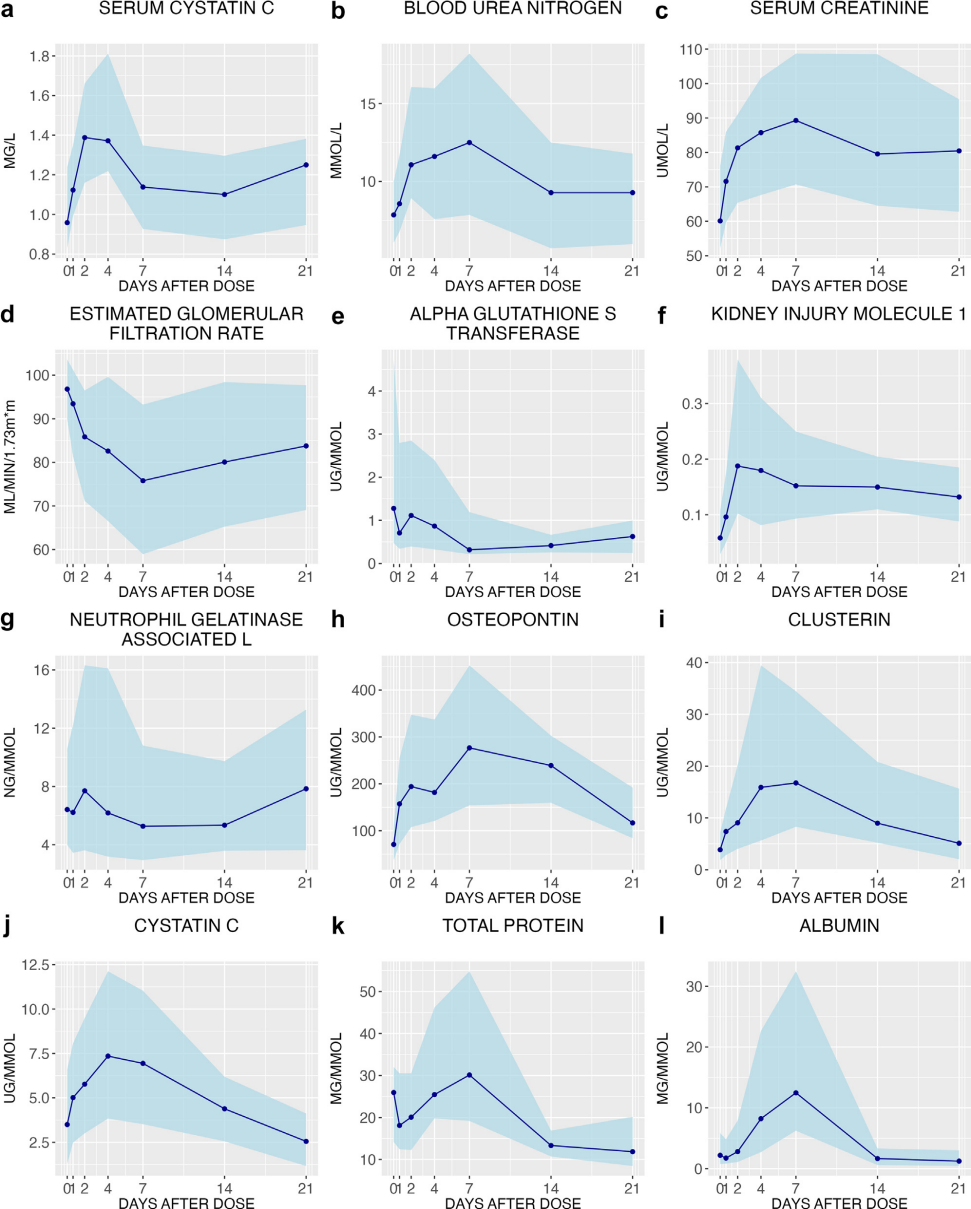
The median time to peak change from baseline for each of the measured urine and serum biomarkers in the cisplatin treated group. Each point represents the median biomarker concentration on the day of sample collection (indexed to urine creatinine to correct for variability of urinary tonicity). The shaded areas represent the upper and lower concentration ranges. Samples were collected pre-dose, 12 hours post cisplatin dose, and on day 1, 2, 4, 7, 14, and 21. Serum biomarkers include: (a) serum cystatin C, (b) blood urea nitrogen, (c) serum creatinine, and (d) estimated glomerular filtration rate. Urinary biomarkers include: (e) alpha glutathione S transferase, (f) kidney injury molecule-1 (KIM-1), (g) neutrophil gelatinase-associated lipocalin (NGAL), (h) osteopontin, (i) clusterin, (j) cystatin C, (k) total protein, and (l) albumin.

Another core objective of the SAFE-T/IMI project was to investigate the time course of median BM values following cisplatin exposure. In Figure 2, we depict the median time to peak concentration for each BM and their upper and lower concentration ranges. Among the BMs, alpha-glutathione-S-transferase exhibited the earliest peak, reaching its median peak concentration on day 1. Urinary NGAL, KIM-1, and serum CYSC followed, with their median peak concentrations occurring on day 2.

### BM Performance at Stage 1 Adjudication (SBMs)

We conducted further analysis to explore the sensitivity of the NBMs in patients identified as having DIKI after stage 1 adjudication of the treated group. Stage 1 adjudication of the Treated cancer group had categorized patients as “DIKI” (*n* = 24), “no-DIKI” (*n* = 71), or “uncertain” (*n* = 7). Patients categorized as uncertain were due to incomplete BM datasets or no consensus between adjudicators and were excluded from analysis because inclusion did not affect the measured outcomes.

**Figure 3:**
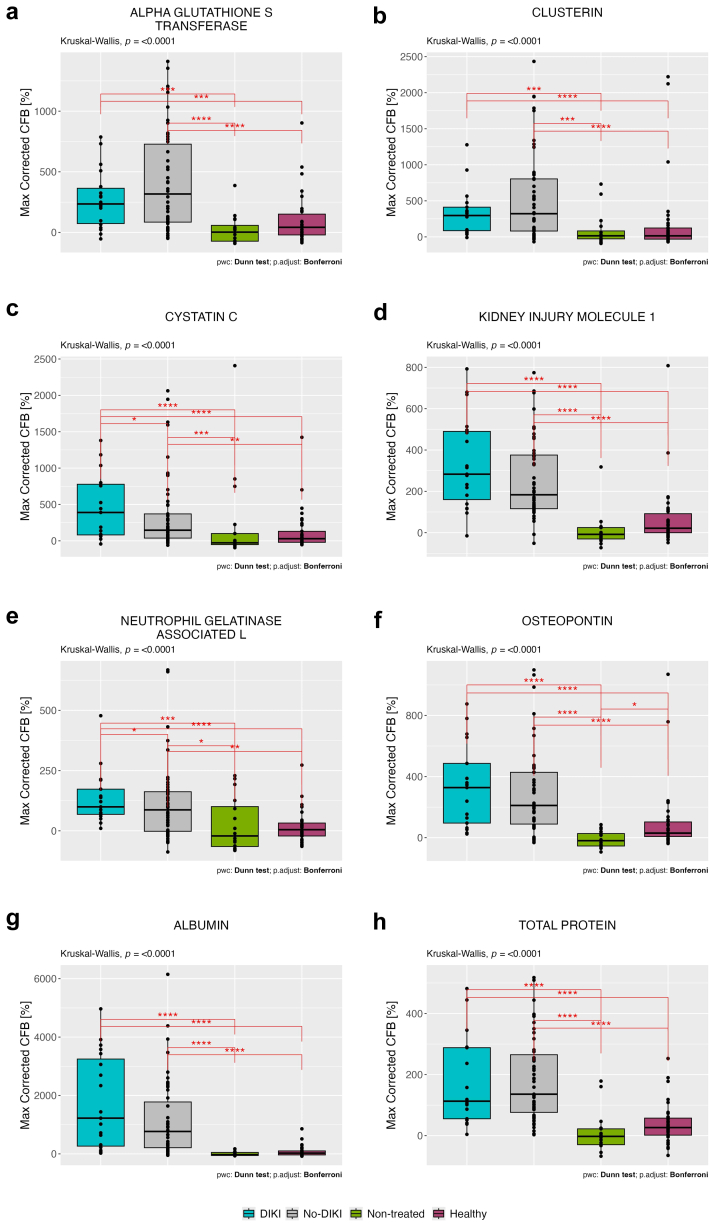
Percent change from baseline in each urinary biomarker corrected for urinary creatinine levels for the stage 1 adjudicated drug-induced kidney injury (DIKI), no-DIKI, healthy, and non-treated groups. The box-and-whisker plots depict the 25th and 75th interquartile ranges, median, and total observed ranges. Lines between groups indicate comparisons made using Dunn's *post hoc* test following a significant Kruskal-Wallis test. Adjustment for the multiple comparisons was performed using the Bonferroni method. Symbols represent significance levels: ∗*P* < 0.05, ∗∗*P* < 0.01, ∗∗∗*P* < 0.001, ∗∗∗∗*P* < 0.0001. There were no differences between DIKI and no-DIKI groups, except cystatin C and neutrophil gelatinase-associated lipocalin. Both DIKI and no-DIKI groups showed significantly greater increases from baseline in all biomarkers compared to healthy and non-treated controls. (a) Alpha glutathione S transferase, (b) clusterin, (c) cystatin C, (d) kidney injury molecule-1 (KIM-1), (e) neutrophil gelatinase-associated lipocalin (NGAL), (f) osteopontin, (g) albumin, and (h) total protein. CFB, change from baseline.

There was a significant difference in all urinary BM changes from baseline between DIKI, no-DIKI, nontreated, and healthy control groups (*P* < 0.0001) (Figure 3). However, on *post hoc* analysis, there was no significant difference between the adjudicated DIKI and no-DIKI groups’ PCFBs. The exceptions were NGAL and urinary CYSC (*P* < 0.05). Furthermore, both the DIKI and no-DIKI groups showed significantly greater PCFBs in all BMs than the healthy and nontreated controls. There were no significant differences between the nontreated cancer control group and healthy control group, except for OPN (*P* < 0.05), which was higher in the healthy control group.

### BM Performance at Stage 2 Adjudication (SBMs and NBMs)

**Figure 4:**
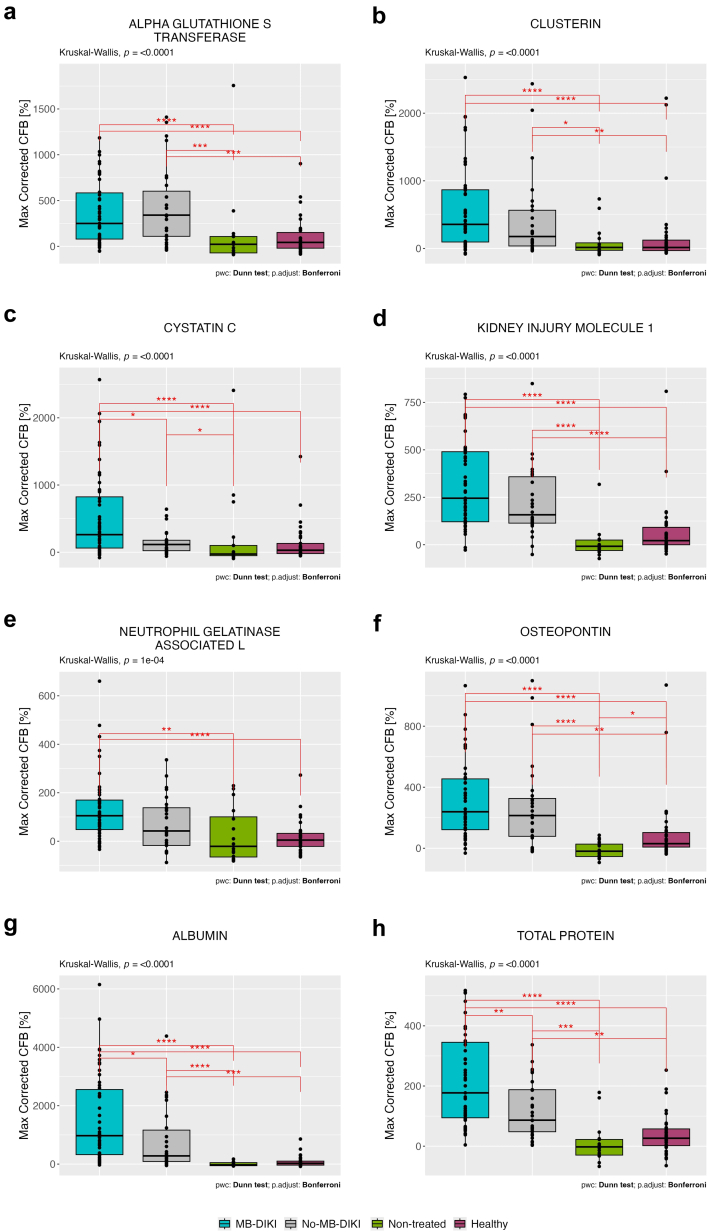
The percent change from baseline in each urinary biomarker corrected for urinary creatinine levels for the stage 2 adjudicated multiple biomarker drug-induced kidney injury (MB-DIKI), no-MB-DIKI, healthy, and non-treated groups. The box-and-whisker plots depict the 25th and 75th interquartile ranges, median, and total observed ranges. Lines between groups indicate comparisons made using Dunn's *post hoc* test following a significant Kruskal-Wallis test. Adjustment for the multiple comparisons was performed using the Bonferroni method. Symbols represent significance levels: ∗*P* < 0.05, ∗∗*P* < 0.01, ∗∗∗*P* < 0.001, ∗∗∗∗*P* < 0.0001. There was a significant difference in change from baseline in all urinary biomarkers between MB-DIKI non-treated and healthy. There was a significant difference in change from baseline in cystatin C, albumin, and total protein in MB-DIKI group compared to no-MB-DIKI group. (a) Alpha glutathione S transferase, (b) CLUSTERIN, (c) cystatin C, (d) kidney injury molecule-1 (KIM-1), (e) neutrophil gelatinase-associated lipocalin (NGAL), (f) osteopontin, (g) albumin, and (h) total protein. CFB, change from baseline.

At stage 2 of adjudication, the treated group was adjudicated using NBMs and SBMs as MB-DIKI (*n* = 63) and no-MB-DIKI (*n* = 32). There was a significant difference in the PCFB in all urinary BMs in the MB-DIKI group compared with the nontreated cancer control and healthy control groups (Figure 4). There was a significant difference in the PCFB in the MB-DIKI group compared with the no-MB-DIKI group for the urinary BMs, CYSC, ALB, and total protein. The median PCFBs for clusterin, KIM-1, NGAL, and OPN were higher in the MB-DIKI group than in the no-MB-DIKI group but did not reach statistical significance.

Most BMs demonstrated good discriminatory capabilities compared with the nontreated cancer group, with AUROC values exceeding 0.8. Notably, ALB, OPN, and KIM-1 exhibited the highest AUROC values of 0.97 (95% CI: 0.93–1.00), 0.96 (95% CI: 0.92–0.99), and 0.96 (95% CI: 0.89–1.00), respectively (Table 3). SCr and eGFR had AUROC values of 0.88 (95% CI: 0.79–0.95) and 0.87 (95% CI: 0.78–0.95), respectively.

**Table 3:** Urinary and serum biomarker accuracy and thresholds for maximum percent change from baseline values in the multiple biomarker DIKI group (MB-DIKI) compared with the nontreated cancer control group

Biomarker	MB-DIKI	Nontreated group	AUROC and 95% CIs	Median BM threshold and 95% CIs	Threshold sensitivity	Threshold specificity
Albumin	62	17	0.97 [0.93–1.00]	112.33 [57.98–284.09]	0.94 [0.82–0.98]	1.00 [0.88–1.00]
Osteopontin	58	17	0.96 [0.92–0.99]	74.69 [31.19–128.80]	0.90 [0.79–0.97]	0.94 [0.88–1.00]
KIM-1	58	17	0.96 [0.89–1.00]	55.56 [55.56–95.25]	0.97 [0.91–1.00]	0.94 [0.82–1.00]
Blood urea nitrogen	68	17	0.95 [0.90,0.99]	15.15 [4.76,36.59]	0.88 [0.78,0.97]	0.94 [0.82,1.00]
Total protein	63	17	0.95 [0.88–1.00]	51.11 [37.75–58.23]	0.97 [0.89–1.00]	0.88 [0.71–1.00]
Serum cystatin C	70	16	0.92 [0.85–0.97]	114.58 [33.33–217.07]	0.91 [0.73–0.97]	0.88 [075–1.00]
Serum creatinine	69	17	0.88 [0.79–0.95]	21.55 [4.11–24.44]	0.78 [0.67–0.91]	0.88 [0.76–1.00]
eGFR	69	17	0.87 [0.78–0.95]	-5.84 [-16.99; -5.09]	0.84 [0.68–0.93]	0.88 [0.71–1.00]
α-GST	58	17	0.84 [0.71–0.94]	114.58 [33.33–217.07]	0.79 [0.64–0.93]	0.82 [0.65–0.97]
Cystatin C	58	17	0.82 [0.68–0.93]	21.07 [−7.38 to 234.55]	0.90 [0.66–0.97]	0.76 [0.53–0.88]
Clusterin	58	17	0.81 [0.66–0.92]	240.00 [27.72–280.89]	0.81 [0.67–0.95]	0.82 [0.59–0.94]
NGAL	58	17	0.73 [0.52–0.89]	21.65 [−23.74 to 121.03]	0.84 [0.59–1.00]	0.65 [0.41–0.82]

AUROC, area under the receiver operating characteristics curve; BM, biomarker; CI, confidence interval; eGFR, estimated glomerular filtration rate; KIM-1: kidney injury molecule-1; NGAL, neutrophil gelatinase-associated lipocalin; α-GST, α- glutathione s-transferase.

Missing data were due to incomplete datasets. Urinary biomarkers were creatinine-corrected. Albumin (mg/mmol), α-GST (μg/mmol), (blood urea nitrogen (mmol/l), clusterin (μg/mmol), eGFR (ml/min per 1.73 m^2^), KIM-1 (μg/mmol), NGAL (ng/mmol), osteopontin (μg/mmol), serum creatinine (mg/dl), serum cystatin c (mg/l), total protein (mg/mmol), urine cystatin c (μg/mmol), α-GST(μg/mmol).

At the maximum PCFB thresholds, the sensitivity was highest for KIM-1 (0.97; 95% CI: 0.91–1.00) and total protein (0.97; 95% CI: 0.89–1.00), whereas the specificity was highest for ALB (1.00; 95% CI: 0.88–1.00). NGAL showed the lowest AUROC (0.73), sensitivity (0.84), and specificity (0.65) among the BMs.

The BM median time to peak in the adjudicated forms was similar between the AKI and MB-DIKI groups; however, the peak median concentration for each BM was lower in the MB-DIKI group than in the AKI group, except for alpha-glutathione-S-transferase. In addition, the median concentrations at each timepoint were lowest in the no-MB-DIKI group for most BMs (Supplementary Figure S1).

## Discussion

The NBMs presented in this study met the SAFE-T/IMI project goals by sensitively measuring cisplatin exposure and enabling timely detection of cisplatin-induced DIKI compared with the SBMs. Notably, alpha-glutathione-S-transferase had the fastest time to peak from baseline compared with all other BMs, whereas KIM-1, OPN, and ALB had the highest accuracy. In addition, the change from baseline in all urinary BMs in both cancer populations was higher in those exposed to cisplatin (treated group) than in the nontreated cancer control group. This is most likely attributed to the direct dose-dependent nephrotoxicity of cisplatin and the damage BM response to injury after exposure, thus supporting the use of these BMs to detect cisplatin exposure. The NBMs response to a nephrotoxin is consistent with other studies which investigated NBMs accuracy and time-dependent changes compared with SBMs.^5^^,^^26^, ^27^, ^28^, ^29^, ^30^ By enabling earlier and sensitive detection, these NBMs could support more timely adjustments in clinical management for patients receiving cisplatin. Tailored interventions, such as modifying the nephrotoxic agent or implementing nephroprotective strategies, may then be employed to prevent further kidney damage.^1^^,^^3^

It should be noted that within the nontreated cancer control group, some patients had been exposed to nephrotoxic chemotherapies, such as oxaliplatin, ifosfomide, 5-flurouracil, and methotrexate (Supplementary Table S2). This may explain the change from baseline and outliers observed in the nontreated group’s BMs.^31^^,^^32^ In addition, there were differences in the types of cancers between the treated and nontreated groups. Within the nontreated group, kidney function was lower compared with the other groups, potentially indicating worse underlying comorbidities, such as diabetes, advanced cancer stage, or cardiovascular disease.

A key strength of this study was the 2-stage adjudication process. During stage 1, which utilized SBMs alone and aligned with the KDIGO AKI criteria, we identified changes from baseline in novel urinary BMs that were not detected using functional BM tools, signifying potential subclinical AKI. When both novel and SBMs were applied, the detection of cisplatin-DIKI improved. Moreover, the time to peak was similar in the DIKI group and MB-DIKI group. This process highlighted the limitations of standard diagnostic methods in identifying the often-overlooked subclinical AKI. The 2-stage adjudication process further demonstrated that integrating NBMs with SBMs provided a more robust diagnostic approach for detecting cisplatin-DIKI. This finding aligns with the 23rd Acute Disease Qualitative Initiative framework, which emphasizes the value of combining damage and functional BMs for more sensitive AKI detection and the differentiation of subphenotypes.^13^^,^^33^

**Figure 5:**
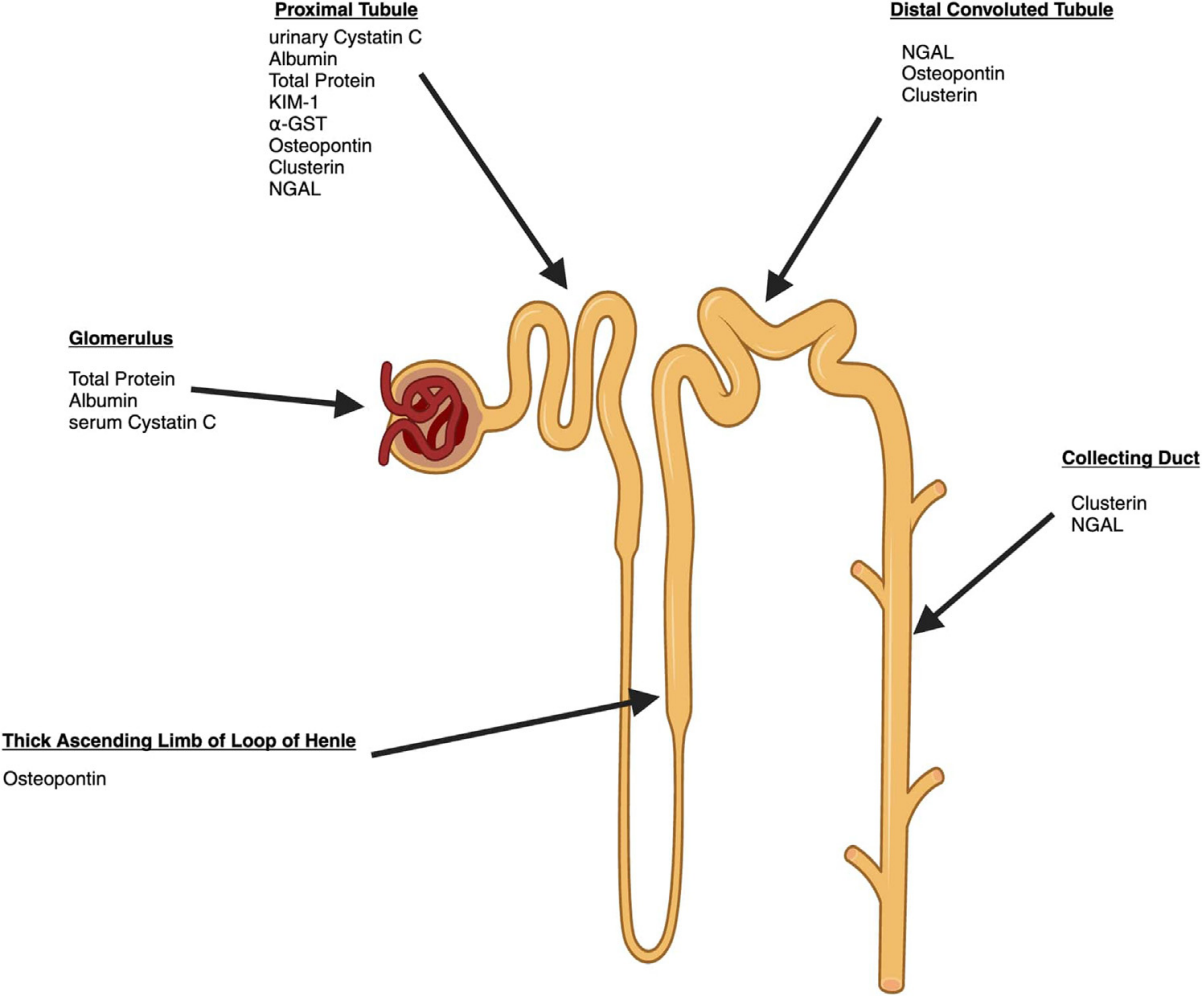
Nephron and the locations at which biomarkers signify injury. Majority of biomarkers are urinary biomarkers, except serum cystatin C. A rise in the biomarkers after nephrotoxin exposure can help localize site of injury and provide information on the pathophysiological process leading to kidney injury. α-GST, alpha-glutathione-S-transferase; KIM-1, kidney injury molecule-1; NGAL, urinary neutrophil gelatinase-associated lipocalin.

The advantage of the NBMs presented in this study lies in their diversity. Each BM represents distinct areas of nephron damage and physiological responses to injury (Figure 5). Consequently, a panel combining these BMs holds promise for more comprehensive identification of nephrotoxicity. This was supported by the qualification letter published by the US Food and Drug Administration, with support from the European Medicines Agency, which outlined the use of a panel of 6 novel urinary BMs in addition to SBMs. The context of use was for phase 1 trials in healthy volunteers, but they also supported the broader exploration of the panel of BMs.^34^

Because this was an exploratory study, several limitations must be highlighted. These include restricting data collection to a single cycle of cisplatin chemotherapy in the treated group, a relatively small sample size, and the use of limited control groups that were not matched for comorbidities or sample timepoint collection. These factors resulted in wider CIs, which included 1 in some cases, complicating the demonstration of the BMs' superiority over standard markers. In addition, using the maximum PCFBs for BM evaluation may introduce bias, because groups with more frequent measurements, such as the treated group with 7 measurement timepoints compared with control groups (2–3 timepoints), might show larger observed changes. Furthermore, the study design did not allow us to control for other causes of AKI. This may have contributed to the BM changes from baseline in the healthy group and nontreated cancer control group. Future studies with larger sample sizes, well-controlled designs, standardized timepoint collections over multiple treatment cycles, and standardized adjudication processes are needed to address these limitations. In addition, leveraging biobanked data could further elucidate the diagnostic value of these NBMs.

Further investigation into a panel of BMs to provide granularity in the complex, heterogeneous syndrome of AKI will move us toward precision medicine and reduce negative outcomes. Moreover, future study designs that investigate changes in BMs throughout each chemotherapy cycle may identify the acute kidney disease population and detect ongoing subclinical AKI.

Cisplatin-induced DIKI is a common complication in hospitals and often a late-stage factor in drug development.^10^^,^^11^^,^^35^ Novel urinary BMs have demonstrated significant potential for the sensitive and early recognition of nephrotoxin exposure and DIKI.^27^^,^^31^ In addition, NBMs provide insight into the underdiagnosed but clinically important subclinical AKI.^1^^,^^13^

## Disclosure

PTM reported consulting for Renibus Therapeutics, Novartis, Alexion, CalciMedica, Bioporto Diagnostics, and Foundation for NIH; grants from Health Research Board, Ireland; and funding to his institution from Abbott. S. Sultana reported stocks Pfizer Stock owned Novartis Stock owned in past 36 months. AstraZeneca Stock owned in past 36 months, stock options. GF also reported stocks with Pfizer as an employee. All the other authors declared no competing interests.

## Supplementary Material

Supplementary File (PDF) SAFE-T Protocol (page 1–10). **Figure S1.** Median time to peak for each BM based on AKI status using standard and modified AKI criteria. **Table S1.** Proportion of cancers in treated and nontreated group. **Table S2.** Cancer treatments for treated and nontreated groups. **Table S3.** Stage gate analysis biomarker list. **Table S4.** Reasons for inclusion/exclusion of stage gate biomarkers. **Table S5.** Assays for biomarkers used. **Table S6.** Assays for biomarkers used. **Table S7.** Demographics for treated and nontreated groups. STROBE Checklist.

## Footnotes

Supplementary File (PDF)
SAFE-T Protocol (page 1–10) **Figure S1.** Median time to peak for each BM based on AKI status using standard and modified AKI criteria. **Table S1.** Proportion of cancers in treated and nontreated group. **Table S2.** Cancer treatments for treated and nontreated groups. **Table S3.** Stage gate analysis biomarker list. **Table S4.** Reasons for inclusion/exclusion of stage gate biomarkers. **Table S5.** Assays for biomarkers used. **Table S6.** Assays for biomarkers used. **Table S7.** Demographics for treated and nontreated groups. STROBE Checklist.

## References

[1] Rodrigues C.E., Endre Z.H. (Feb 2023). Definitions, phenotypes, and subphenotypes in acute kidney injury-Moving towards precision medicine. Nephrology (Carlton).

[2] Mehta R.L., Awdishu L., Davenport A. (2015). Phenotype standardization for drug-induced kidney disease. Kidney Int.

[3] Ostermann M., Bellomo R., Burdmann E.A. (Aug 2020). Controversies in acute kidney injury: Conclusions from a Kidney Disease: Improving Global Outcomes (KDIGO) Conference. Kidney Int.

[4] Pabla N., Dong Z. (2008). Cisplatin nephrotoxicity: Mechanisms and renoprotective strategies. Kidney Int.

[5] Jelinek M.J., Lee S.M., Wyche Okpareke A. (2018). Predicting acute renal injury in cancer patients receiving cisplatin using urinary neutrophil gelatinase-associated lipocalin and cystatin C. Clin Transl Sci.

[6] Lim Y.J., Xiu S.G., Kuruvilla M.S. (2023). Metabolomic identification of predictive and early biomarkers of cisplatin-induced acute kidney injury in adult head and neck cancer patients. Br J Clin Pharmacol.

[7] Jain A., Huang R., Lee J. (2021). A Canadian study of cisplatin metabolomics and nephrotoxicity (Accent): A clinical research protocol. Can J Kidney Health Dis.

[8] Odutayo A., Wong C.X., Farkouh M. (2017). AKI and long-term risk for cardiovascular events and mortality. J Am Soc Nephrol.

[9] Coca S.G., Singanamala S., Parikh C.R. (2012). Chronic kidney disease after acute kidney injury: A systematic review and meta-analysis. Kidney Int.

[10] Kulkarni P. (2021). Prediction of drug-induced kidney injury in drug discovery. Drug Metab Rev.

[11] Griffin B.R., Faubel S., Edelstein C.L. (2019). Biomarkers of drug-induced kidney toxicity. Ther Drug Monit.

[12] Kidney Disease: Improving Global Outcomes (KDIGO) Acute Kidney Injury Work Group (2012). KDIGO clinical practice guideline for acute kidney injury. Kidney Int Suppl.

[13] Ostermann M., Zarbock A., Goldstein S. (2020). Recommendations on acute kidney injury biomarkers from the Acute Disease Quality Initiative Consensus Conference: A consensus statement. JAMA Network Open.

[14] Redahan L., Murray P.T. (2018). Novel biomarkers of drug-induced kidney injury. Clin Pharmacol Ther.

[15] Holditch S.J., Brown C.N., Lombardi A.M., Nguyen K.N., Edelstein C.L. (2019). Recent advances in models, mechanisms, biomarkers, and interventions in Cisplatin-Induced acute kidney injury. Int J Mol Sci.

[16] Côté J.-M., Authier R., Ethier I. (2022). Clinical implementation of NGAL testing to improve diagnostic assessment of AKI episodes in a Canadian Center. Can J Kidney Health Dis.

[17] Matheis K., Laurie D., Andriamandroso C. (2011). A generic operational strategy to qualify translational safety biomarkers. Drug Discov Today.

[18] Helmersson-Karlqvist J., Arnlov J., Larsson A. (2013). Day-to-day variation of urinary NGAL and rational for creatinine correction. Clin Biochem.

[19] Tang K.W., Toh Q.C., Teo B.W. (2015). Normalisation of urinary biomarkers to creatinine for clinical practice and research--when and why. Singapore Med J.

[20] Wen Y., Thiessen-Philbrook H., Moledina D.G. (2022). Considerations in controlling for urine concentration for biomarkers of kidney disease progression after acute kidney injury. Kidney Int Rep.

[21] Lee J.W., Devanarayan V., Barrett Y.C. (2006). Fit-for-purpose method development and validation for successful biomarker measurement. Pharm Res.

[22] Lee J.W., Hall M. (2009). Method validation of protein biomarkers in support of drug development or clinical diagnosis/prognosis. J Chromatogr B Analyt Technol Biomed Life Sci.

[23] Choi Y.-K., Johnson W.O., Collins M.T., Gardner I.A. (2006). Bayesian inferences for receiver operating characteristic curves in the absence of a gold standard. J Agric Biol Environ Stat.

[24] R Core Team (2024). R: A Language and Environment for Statistical Computing.

[25] SAS Institute Inc (2013). SAS/STAT® 9.4 User’s Guide.

[26] Shinke H., Masuda S., Togashi Y. (2015). Urinary kidney injury molecule-1 and monocyte chemotactic protein-1 are noninvasive biomarkers of cisplatin-induced nephrotoxicity in lung cancer patients. Cancer Chemother Pharmacol.

[27] Szumilas D., Owczarek A.J., Brzozowska A., Niemir Z.I., Olszanecka-Glinianowicz M., Chudek J. (2024). The value of urinary NGAL, KIM-1, and IL-18 measurements in the early detection of kidney injury in oncologic patients treated with cisplatin-based chemotherapy. Int J Mol Sci.

[28] de Geus H.R., Fortrie G., Betjes M.G., van Schaik R.H., Groeneveld A.J. (2013). Time of injury affects urinary biomarker predictive values for acute kidney injury in critically ill, non-septic patients. BMC Nephrol.

[29] Ibrahim M.E., Chang C., Hu Y. (2019). Pharmacokinetic determinants of cisplatin-induced subclinical kidney injury in oncology patients. Eur J Clin Pharmacol.

[30] Waikar S.S., Mogg R., Baker A.F. (2025). Urinary kidney injury biomarker profiles in healthy individuals and after nephrotoxic and ischemic injury. Clin Pharmacol Ther.

[31] Abdelsalam M., Elmorsy E., Abdelwahab H. (2018). Urinary biomarkers for early detection of platinum based drugs induced nephrotoxicity. BMC Nephrol.

[32] Karam S., Rosner M.H., Sprangers B., Stec R., Malyszko J. (2024). Cancer therapy in patients with reduced kidney function. Nephrol Dial Transplant.

[33] Vanmassenhove J., Van Biesen W., Vanholder R., Lameire N. (2019). Subclinical AKI: Ready for primetime in clinical practice?. J Nephrol.

[34] Center for Drug Evaluation and Research (2018). Qualification Determination Letter. US Food And Drug Administration.

[35] Karimzadeh I., Barreto E.F., Kellum J.A. (2023). Moving toward a contemporary classification of drug-induced kidney disease. Crit Care.

