# Corrigendum to “Early and Sensitive Detection of Cisplatin-Induced Kidney Injury Using Novel Biomarkers” [*Kidney International Reports* Volume 10, Issue 4, April 2025, Pages 1175-1187]

**DOI:** 10.1016/j.ekir.2025.103723

**Published:** 2025-12-09

**Authors:** Michael Strader, Gary Friedman, Xavier Benain, Nunzio Camerlingo, Stefan Sultana, Shiran Shapira, Nadir Aber, Patrick T. Murray

**Affiliations:** 1 Department of Medicine, School of Medicine, University College Dublin, Dublin, Ireland; 2 Global Biometrics and Data Management, Pfizer, Inc., Cambridge, Massachusetts, USA; 3 Biostastics and Programming, Sanofi S.A., Paris, France; 4 Patient Safety Center of Excellence, AstraZeneca PLC, Cambridge, UK; 5 Health Promotion Center and Integrated Cancer Prevention Center, Tel Aviv Sourasky Medical Center and Tel Aviv University, Tel Aviv, Israel

The authors regret that there were errors in the published article in which the corrections will be outlined here. Two areas of concern are (1) (a) Incorrect labeling of a published original figure in the paper; and (b) similarly incorrect labeling of the first Supplementary figure; and (2) details of the design-phase study sample size estimation.

Specific details of the errors are as follows:

**1. Figure Legend Errors:**

(a) The first line in Figure 2 on Page 1181 is: “The median time to peak change from baseline for each of the measured serum and urine biomarkers in the cisplatin treated group.” Rather, these are time profile plots of median concentrations (with upper and lower ranges) of serum and urine markers. Although the X and Y axes are correctly labelled (time and concentration, respectively) and accurately described as median biomarker concentrations in the remainder of the legend, the title is inaccurate.

This should have read, “**Time profile plots of median BM concentrations** for each of the measured serum and urine biomarkers in the cisplatin treated group.”

Similarly, the associated text in the paper on page 1180 states that “In Figure 2, we depict the **median time to peak concentration** for each BM and their upper and lower concentration ranges.” The preceding sentence correctly states that “a core objective....to investigate the time course of median BM values following cisplatin exposure”, and inspection of these profiles reveals variable time to peak median values, which are correctly described in the remainder of the paragraph.

This should have read, “In Figure 2, we depict **the time course of median BM** values for each BM and their upper and lower concentration ranges.”

(b) Supplementary Figure S1**: on page 15 of the supplement**. Currently labeled: **“Median time to peak based per AKI group classification.”**

This label should have read, **“Time course of median BM values per AKI group classification.”**

Supplementary Figure S1 is also referenced in the text of the main paper, on page 1184, at the end of the Results section:

“The BM median time to peak in the adjudicated forms was similar between the AKI and MB-DIKI groups; however, the peak median concentration for each BM was lower in the MB-DIKI group than in the AKI group, except for alpha-glutathione-S-transferase. In addition, the median concentrations at each timepoint were lowest in the no-MB-DIKI group for most BMs (Supplementary Figure S1).”

These statements accurately reflect the time course of median BM values, and don’t require changes.

Finally, the listing of Supplementary material on page 1186 includes: “Figure S1. **Median time to peak** for each BM based on AKI status using standard and modified AKI criteria.”

This should have read, **“Time course of median BM values per AKI group classification.”**

In summary, although the paper currently describes the data with sufficient accuracy, we request correction of a few phrases to improve the Figures, legends, and associated text.

2. Sample Size Estimation:

The method for sample size estimation reported in paragraph 2 of the Statistical Analysis section on page 877 was not the source of the sample sizes reported in the paper. The Choi method (a non-gold standard, Bayesian approach) as described was proposed in the original, *draft* Statistical Analysis Plan (SAP); we mistakenly understood that this was the approach used by the Consortium to determine the sizes of the cohorts. However, as detailed in the full study protocol (**attached**; which was truncated in the submitted and published Appendix, deleting the sample size and statistical analysis sections and other subsequent sections on pages 14-19), it is clear that sample size estimates for this ***exploratory study*** were based on expectation of a 20% incidence of cisplatin-induced AKI in 100 cisplatin-treated subjects, and a planned enrolment of 20 healthy control subjects and 20 cancer controls (treated with non-nephrotoxic regimens) for comparison; as detailed in Section 9.1 of the protocol.

The protocol also contained a provision for ***interim*** analysis: “The sample size of the cisplatin-treated and the control cohorts may be adjusted during the course of the study based on interim analyses of novel biomarker patterns as well as the incidence of AKI as defined by BUN and serum creatinine changes in the cisplatin cohort.”

Finally, **Section 9.4** of the protocol also mentions interim analysis:

**“Interim Analysis”**

An interim analysis of the biomarker endpoints will take place after the data from 30 cisplatin cohort patients are entered on to the database and are available for analysis.”

**2. (a) Paragraph 2 of the Statistical Analysis section on page 877 should be replaced with the following paragraph, which is taken directly from the study protocol, apart from the opening and closing sentences:**

“Sample size estimates for this exploratory phase study were based upon published AKI rates of 15%-25% (circa 2012–2014) following cisplatin treatment. The maximum sample size chosen for this multiple cohort observational study is 140 patient/subjects comprising:

▪ 100 patients in the cisplatin cohort to have a minimum of 20 subjects who show post-cisplatin BUN and serum creatinine changes that qualify as AKI.
▪ 20 patients in the patient control cohort.
▪ 20 subjects in the volunteer control cohort.

A sample size of 100 patients in the cisplatin cohort and 20 patients/subjects in each control cohort was chosen to enable a reliable assessment of the form of the statistical distribution for baseline values for each novel BM. In addition, sample sizes of 20 cisplatin patients with AKI and 20 patients/subjects in each control cohort were selected to enable an exploratory comparison between cohorts to detect patterns of difference in the BM profiles over time. The protocol contained a provision for interim analysis and potential sample size adjustments of the cisplatin-treated and the control cohorts during the course of the study based on analyses of novel biomarker patterns as well as the incidence of AKI, but no interim analysis for sample size adjustment was done.”

The authors apologize for any inconvenience.

DOI of original article: https://doi.org/10.1016/j.ekir.2025.01.035

