# Supplementary material for: Early and Sensitive Detection of Cisplatin-Induced Kidney Injury Using Novel Biomarkers
Source: Kidney Int Rep. 2025 Feb 5;10(4):1175–87. doi: 10.1016/j.ekir.2025.01.035 (PMC12034874; doi:10.1016/j.ekir.2025.01.035)
Supplement: Supplementary File (PDF) — SAFE-T Protocol (page 1–10). Figure S1. Median time to peak for each BM based on AKI status using standard and modified AKI criteria. Table S1. Proportion of cancers in treated and nontreated group. Table S2. Cancer treatments for treated and nontreated groups. Table S3. Stage gate analysis biomarker list. Table S4. Reasons for inclusion/exclusion of stage gate biomarkers. Table S5. Assays for biomarkers used. Table S6. Assays for biomarkers used. Table S7. Demographics for treated and nontreated groups. STROBE Checklist. [file mmc1.pdf]

Supplementary Material

Supplementary Material 1

1.1 Study Aim

The primary focus of the study is to assess novel markers of renal safety. Stored samples from this study will also be available for future research in renal safety markers as well as those of other organ systems.

The study will enrol cancer patients who are scheduled to receive high dose cisplatin as monotherapy or as part of a combination chemotherapy regimen. The cisplatin treatment will be administered as part of the standard of care management of these patients' cancer; there will not be any change to patients' cancer treatment as a result of participating in this study. Control patients and healthy volunteers will be recruited for comparison purposes. Blood and urine samples will be collected at baseline and at various time-points after the first administration of cisplatin to assess the time-course of any changes in the various novel renal injury biomarkers and compare these with BUN and serum creatinine changes.

2. STUDY OBJECTIVES AND ENDPOINTS

2.1 Objectives

- To collect blood and urine samples in cisplatin-treated patients and control subjects.
- To characterise the within- and between-subject baseline variability of novel biomarkers relative to BUN/ serum creatinine in these populations.
- To compare the patterns of novel biomarker changes relative to BUN/ serum creatinine following cisplatin treatment. These data will be used to:
    - select candidate biomarkers to progress to the confirmatory stage of biomarker qualification and establish cut-off values for these biomarkers.
    - determine the optimum method of expressing (corrected vs. uncorrected) each novel urinary marker relative to urinary creatinine for subsequent studies.
    - characterise the time course of biomarker changes to optimise the study design and sample collection time-points of confirmatory studies.

2.2 Endpoints

- For each novel serum and urinary biomarker, as well as for BUN and serum creatinine, the following will be determined:
    - Maximum change from baseline.
    - Time to maximum change from baseline.
    - Once cut-off values are determined, time to abnormal change.
    - Time to return to baseline (or to return to normal range).
    - Mean change over period of observation (AUEC analysis)

- Novel urinary biomarkers will be assessed in the following ways in the various analyses of interest:
    - corrected for urinary creatinine concentration, and
    - uncorrected biomarker concentration.

3. STUDY DESIGN

This is a prospective, non-randomised, longitudinal, case control study. Patients with cancer who are scheduled to receive high dose cisplatin as part of their standard of care treatment (e.g. non-small cell lung cancer, head & neck cancer) will be enrolled in this study. Control patients with similar cancers who have not had recent major surgery or treatment with nephrotoxic drugs will be enrolled as a control patient population. A cohort of healthy subjects will be enrolled as a separate healthy control population.

Subjects will have a screening assessment where their diagnosis and eligibility for enrolment is confirmed. The study will be explained to them and subjects willing to participate will be asked to give informed consent. Unless documented by clinical chemistry laboratory results in the previous month, subjects will have a blood sample taken for BUN and serum creatinine to document baseline renal function and confirm they meet the criteria for the study. Eligible patients scheduled to receive cisplatin will have blood and urine samples taken pre-dose on the day of first cisplatin administration and at intervals over the next 3 weeks. Control subjects (cancer patients and healthy volunteers) will attend the clinic to give blood and urine samples at two visits that are 2 days (+/- 1 day) apart.

Enrolled subjects will have blood and urine samples collected for novel biomarkers at the following time-points:

Cisplatin-treated group:

- Pre-dose on day of 1st cycle cisplatin chemotherapy administration (baseline)
- Prior to leaving the clinic following cisplatin administration (or at approximately 8 - 12 hours post-dose for in-patients)
- 24 hours post-dose
- 2 days post-dose
- 4 days (+/- 1 day) post-dose
- 7 days (+/- 1 day) post-dose
- 14 days (+/- 3 days) post-dose
- 21 days (+/- 7 days) post-dose

Control groups (cancer patients and healthy volunteers):

- Visit 1 (within 3 weeks of Screening: this may be the Screening visit after subjects have given their informed consent. In this case, for those subjects who are subsequently found not to qualify for inclusion after lab results are available, stored samples will be discarded and not included in any analysis. Any subjects not included after Screening will not count towards the number of enrolled subjects for the relevant cohort even if samples are taken and later discarded.)
- Visit 2 (2 days [+/- 1 day] after Visit 1)

Note that apart from samples taken in the cisplatin-treated patients in the 12 hour period after cisplatin infusion, all blood and urine samples will be collected in the morning. All subjects will be asked to attend the clinic within a 2-hour window (chosen after consultation between the Investigator and the subject) at each visit as far as possible.

Table 1: Schedule of Activities for cisplatin-treated group of patients

**Cisplatin-treated patient group**	Screening	Pre-dose	<12 hrs	24 hrs	2 days	4 days	7 days	14 days	21 days
Informed consent	x								
Blood sample (5 mL) for clinical chemistry	x	x	x	x	x	x	x	x	x
Urine sample (10 mL) for urinalysis	x	x	x	x	x	x	x	x	x
Check subject eligibility	x								
Review adverse events and concomitant medications	x	x	x	x	x	x	x	x	x
Enrol subject		x							
Blood sample (10 mL) for serum[1]		x	x	x	x	x	x	x	x
Blood sample (10 mL) for plasma[2]		x					x		
Urine sample (40 mL)[1]		x	x	x	x	x	x	x	x
Ensure patient has oncology follow-up visit									x

[1]To analyse for DIKI biomarkers as listed in Table 3.
[2]A plasma sample will be stored for future use for novel renal and other organ safety biomarkers.

Table 2: Schedule of Activities for patient controls

**Patient control group n=20**	Screening [2]	Visit 1[2]	Visit 2 (2 days post V1)
Informed consent	x		
Blood sample (5 mL) for clinical chemistry	x	x	x
Urine sample (10 mL) for urinalysis	x	x	x
Check subject eligibility	x		
Review adverse events and concomitant medications	x	x	x
Enrol subject		x	
Blood sample (10 mL) for serum[1]		x	x
Blood sample (10 mL EDTA) for plasma		x	x
Urine sample (40 mL) [1]		x	x

[1]To analyse for DIKI biomarkers as listed in Table 3.
[2]These visits may be combined if subject gives informed consent

Table 3: Schedule of Activities for healthy control group

**Healthy control group n=20**	Screening [2]	Visit 1[2]	Visit 2 (2 days post V1)	Visit 3 (14 to 28 d post V1)
Informed consent	x			
Blood sample (5 mL) for clinical chemistry	x	x	x	x
Urine sample (10 mL) for urinalysis	x	x	x	x
Check subject eligibility	x			
Review adverse events and concomitant medications	x	x	x	x
Enrol subject		x		
Blood sample (10 mL) for serum[1]		x	x	x
Blood sample (10 mL EDTA) for plasma		x	x	x
Urine sample (40 mL) [1]		x	x	x

[1]To analyse for DIKI biomarkers as listed in Table 3.
[2]These visits may be combined if subject gives informed consent

This is an observational study. All patients in this study will receive treatment as per standard of care for their conditions. Patients in this study that develop BUN or serum creatinine changes following cisplatin treatment that qualify as acute kidney injury will be managed according to standard hospital treatment protocols.

4. SUBJECT SELECTION

4.1 Inclusion Criteria

All subjects

- Males and females ≥18 years of age.

Cisplatin patients

- Patients with documented cancer (e.g. head & neck or similar) who are scheduled to receive 1st cycle high dose cisplatin therapy (≥65 mg/ m^2/ cycle).

Control subjects

- Patients with documented cancer (similar to patients due to be treated with cisplatin) who have received or are scheduled to receive non-nephrotoxic treatment modalities.
- A further group of healthy control subject will be enrolled for comparison.

4.2 Exclusion Criteria

All subjects

- Chronic kidney disease defined by microalbuminuria (>30 mcg/ mg urinary creatinine) or eGFR <60 mL/min/1.73m^2.
- Regular co-administration of any of the following within 7 days prior to screening until the last sample collection time-point.
  - creatine supplements
  - drugs known to alter tubular secretion of creatinine (e.g. trimethoprim, cimetidine).
  - Non-steroidal anti-inflammatory drugs (ibuprofen, diclofenac, naproxen; occasional use allowed).
- Major surgery from 1 month prior to screening until the last sample collection time-point.
- *NOTE*: patients needing to take diuretic, ACEi or ARB drugs may be enrolled and continue on these therapies but these patients will be analysed separately.
- Inability to comprehend, or unwillingness to follow, the study requirements including attendance at out-patient clinic visits and participation in laboratory testing as called for by the protocol.

4.3 Life Style Guidelines

Subjects should avoid strenuous exercise during the 24 hours preceding each biosample collection. Walking, moderate cycling and similar activities are allowed.

5. STUDY TREATMENTS

5.1 Allocation to Treatment

This is an observational study. All patients in this study will receive the standard of care protocol of treatment for their specific malignancy. Healthy subjects will not receive any treatment. Subjects will be enrolled into the study based on whether they are scheduled to receive high dose cisplatin therapy (cisplatin group), non-nephrotoxic therapy (control cancer group) or no treatment (healthy control group).

5.2 Drug Supplies

Standard of care treatment will be provided by the hospital or other institutions where the patients are being treated.

5.3 Concomitant Medication

If any patients require treatment with drugs that are known to be nephrotoxic (e.g. aminoglycoside antibiotics, chronic use NSAIDs) or that interfere with creatinine secretion (e.g. trimethoprim, cimetidine), they will be withdrawn from the study. Any samples collected before they receive any such medications will be included in any pooled analyses of data.

5.4 Rescue/Salvage Therapy

Patients who develop signs or lab test changes that qualify as acute kidney injury will be managed according to the standard protocols of treatment for that institution. This includes both active management and longer-term follow-up of these patients.

The patients' cancer therapy (based on response/non-response to cisplatin for example) will be managed according to that institutions treatment policy for each specific cancer.

6. STUDY PROCEDURES

6.1 Screening

Subjects eligible for the study will have the nature and purpose of the study explained to them by the investigator. They will be provided a written copy of the informed document for the study and given sufficient time to consider the study's implications (e.g. sample collection schedule) before deciding to participate.

The subjects' medical history, prior and concurrent medications, and demographics will be documented. A full physical examination will be done, including blood pressure, body weight and height. A blood sample for BUN and serum creatinine will be taken unless documented by results are available in the previous month. An urine sample will be obtained for routine urinalysis. Eligibility will be determined by checking the inclusion/exclusion criteria.

6.2 Study Visits

Cisplatin treatment group

The cisplatin treatment group of subjects will have blood and urine samples collected as described below. Details of samples collected are given in Section 7.

- Pre-dose blood and urine sample collection before 1st dose of cisplatin chemotherapy.

Patients will then receive the cisplatin infusion as per the standard treatment protocol for their institution. The date and time of starting cisplatin infusion will be recorded in the Case Record Form.

- Blood and urine sample collection within first 12 hours post-1st dose of cisplatin chemotherapy.

Patients who attend for cisplatin treatment on an outpatient basis will have the post-dose set of blood and urine samples collected just before going home. The time of this sample collection will be recorded in the Case Record Form.

For patients who are receiving cisplatin as in-patients, this first post-dose sample should be taken approximately 8 to 12 hours after the start of the cisplatin infusion.

For the 3 weeks following the first cisplatin infusion, patients will attend the clinic on the days described below. Samples should be collected when they arrive in the clinic. If a patient is unable to produce a urine specimen on arrival at the clinic, he/she should be given some fluids to drink in order to produce the sample. As far as possible, clinic visits should be in the morning and should be arranged such that they are within a 2 hour window that is convenient for the patient and clinic staff.

Blood and urine sample collections at:
- 24 hours post-dose.
- 2 days (i.e. 48 hours) post-dose.

Patients should make every effort to attend to give samples at both 24 and 48 hours post-dose. If patients are not able to attend for either time-point, they should be withdrawn from the study and no further samples collected.

- 4 days (+/- 1 day) post-dose.
- 7 days (+/- 1 day) post-dose.
- 14 days (+/- 3 days) post-dose.
- 21 days (+/- 7days) post-dose.

Patients who miss an appointment to give blood and urine samples should be contacted to see if the visit can be rescheduled. If visits cannot be rescheduled and a patient misses more than 2 visits, they should be withdrawn from the study and no further samples collected.

Control groups

Control subjects (non-cisplatin treated cancer patients, healthy volunteers) will have blood and urine samples collected as described below. Note that for cancer control group patients who are receiving inpatient treatment, morning blood and urine will be collected in the hospital or clinic they are being treated in. Details of samples collected are given in Section 7.

Blood samples and a urine sample will be collected when subjects arrive in the clinic. If a subject is unable to produce a urine specimen on arrival at the clinic, he/she should be given some fluids to drink in order to produce the sample. As far as possible, clinic visits should be in the morning and should be arranged such that they are within a 2 hour window that is convenient for the patient and clinic staff.

Samples should be provided on:

- Visit 1: this should be within 3 weeks of the Screening assessment (see previous regarding combining Screening and Visit 1).
- Visit 2: this should 2 days (+/- 1 day) after Visit 1.

6.3 Follow-up Visit

Since this is an observational study, there will be no follow up visit. Cancer patients (both cisplatin-treated group and control patients) will be followed up and treated according to the standard of care protocol of their hospital or institution. The investigator should check that patients have an appointment for their next cancer treatment or clinic visit as appropriate.

6.4 Subject Withdrawal

Subjects can withdraw from the study at any time. There will be no study follow up visit and cancer patients will revert to the usual hospital or clinic visit schedule as per their standard of care.

7. SAMPLE COLLECTION & HANDLING

10 mL blood (serum) and 40 mL urine samples will be collected at each visit for the following kidney biomarkers:

Table 3: Novel biomarkers to be measured in this study

Type of biomarker	Biomarker name*	Main significance
Functional biomarkers	Microalbumin/Albumin	Marker of impaired proximal tubular reabsorption
	α-1 microglobulin	Marker of impaired proximal tubular reabsorption and indirectly of glomerular injury
	Cystatin C	Evaluation of glomerular filtration rate (serum)<br>Marker of impaired proximal tubular reabsorption and indirectly of glomerular injury(urine)
	Urinary creatinine	Marker of impaired proximal tubular reabsorption and indirectly of glomerular injury(urine)
	Retinol Binding Protein-4 (RBP-4)	Marker of impaired proximal tubular reabsorption
Tissue injury leakage markers	N-acetyl-β-D-glucosaminidase (NAG)	Marker of proximal tubular injury
	Glutathione-S-transferase-α (GST-α)	Marker of proximal tubular injury
	Glutathione-S-transferase-π (GST-π)	Marker of distal tubular injury
	Liver-type fatty acid binding protein (L-FABP)	Marker of proximal tubular injury
	Collagen IV	Marker of glomerular injury
	Podocin	Marker of glomerular injury
	Nephrin	Marker of glomerular injury
	Aquaporin-2	Marker of collecting duct injury
	Calbindin D28	Marker of injury to distal regions of nephron and collecting ducts
Tissue injury response markers	Kidney injury molecule-1 (KIM-1)	Marker of proximal tubular injury/regeneration
	Clusterin	Marker of tubular injury/regeneration (no apparent specific nephronal localization)
	Neutrophil gelatinase associated lipocalin (NGAL)	Marker of tubular (mainly proximal) injury

Type of biomarker	Biomarker name*	Main significance
	Trefoil Factor 3 (TFF3)	Marker of proximal tubular injury
	Osteopontin	Marker of injury to distal regions of nephron
	Tissue inhibitor of metalloproteinase-1 (TIMP-1)	Marker of interstitial fibrosis and tubular injury
	Connective Tissue Growth Factor (CTGF)	Marker of interstitial fibrosis
	Interleukin-18 (IL-18)	Marker of inflammation
	Monocyte chemoattractant protein-1 (MCP-1)	Marker of inflammation

* All biomarkers will be assessed in urine and Cystatin C and NGAL will additionally be assessed in plasma/serum.

A 10 mL EDTA blood sample will be taken at the baseline and Day 7 visits only. The plasma obtained from these samples will be stored for future use for novel renal and other organ safety biomarkers.

Additional the following parameters will be assessed in the 5 ml blood plus 10 ml urine for routine diagnostics.

Blood Chemistry	Total protein, Albumin, Creatinine, Urea<br>Sodium, Chloride, Calcium, Phosphate, Potassium, Magnesium
Urinalysis	U-Kreat; U-Urea; Na, K, U-Protein, U-Albumin (Midstream)

Table 4: Type of Samples and Approximate Amount of Blood and Urine to be collected from Each Subject

**Test**	**Sample volume (mL)**	**Number of visits sampled**		**Total volume (mL)**	
		**Cisplatin group**	**Control groups**	**Cisplatin group**	**Control groups**
Routine laboratory test	5	9	3	45	15
DIKI serum sample	10	8	2	80	20
DIKI ETDA blood	10	2	2	20	20
**Total blood volume**	**-**	**-**	**-**	**145**	**55**
Routine urinalysis	10	9	3	90	30
DIKI urine sample	40	8	2	320	80
**Total urine volume**	**-**	**-**	**-**	**410**	**110**

Total Blood Loss

Approximately 145 mL whole blood will be drawn in patients receiving cisplatin treatment and approximately 55 ml whole blood will be drawn in control subjects (healthy volunteers and cancer control patients).

Sample Handling Procedures

Details on blood and urine sample handling and storage at the site as well as shipment conditions for frozen samples are detailed in a separate sample handling SOP.

8. ADVERSE EVENT REPORTING

8.1 Definition of Adverse Events

An Adverse Event (AE) is any untoward medical occurrence in a subject or clinical investigation subject administered a pharmaceutical product and which does not necessarily have to have a causal relationship with the intervention. An AE can therefore be any unfavourable and unintended sign (including an abnormal laboratory finding, for example), symptom, or disease temporally associated with the use of a pharmaceutical product, whether or not considered related to the study. Pre-existing conditions that worsen during a study are to be reported as AEs.

8.2 Reporting Period

All clinical adverse events (AEs) encountered during the clinical study will be recorded on AE source documents and retained at the Investigator site.

8.3 Serious Adverse Events

A serious adverse event (SAE) is any experience that suggests a significant hazard, contraindication, side effect or precaution. An SAE must fulfill at least one of the following criteria:

- is fatal (results in the outcome death)
- is life-threatening
- required in-patient hospitalization or prolongation of existing hospitalization
- results in persistent or significant disability/incapacity
- is a congenital anomaly/birth defect
- is medically significant or requires intervention to prevent one or other of the outcomes listed above

Serious Adverse Events unrelated to the study procedure and /or underlying conditions, must be collected and reported during the study and up to 15 days after the last visit.

8.4 Severity Assessment

All clinical adverse events (AEs) encountered during the clinical study will be recorded on AE source documents and retained at the Investigator site. Intensity of AEs will be graded on a four–point scale (mild, moderate, severe and life threatening). A description of scales can be found below.

- Mild discomfort noticed but no disruption of normal daily activity
- Moderate discomfort sufficient to reduce or affect daily activity
- Severe inability to work or perform normal daily activity
- Life Threatening represents an immediate threat to life

Relationship of the AE to the study procedure or prescribed medications should also be assessed. Details can be found below.

8.5 Causality Assessment

Relationship of the AE to the study procedure or prescribed medications should be assessed as probable, possible, remote or unlikely.

PROBABLE (must have first three)

This category applies to those AEs that are considered, with a high degree of certainty, to be related to the study procedures. An AE may be considered probable, if:

1. It follows a reasonable temporal sequence of the study procedures.
2. It cannot be reasonably explained by the known characteristics of the subject's clinical state, environmental or toxic factors, or other modes of therapy administered to the subject.
3. It disappears or decreases on cessation or reduction of the study procedures.
4. It follows a known pattern of response to the suspected study procedure.
5. It reappears upon rechallenge.

POSSIBLE (must have first two)

This category applies to those AEs in which the connection with the study procedures appears unlikely but cannot be ruled out with certainty. An AE may be considered possible if, or when:

1. It follows a reasonable temporal sequence of the study procedures.
2. It may have been produced by the subject's clinical state, environmental or toxic factors, or other modes of therapy administered to the subject.
3. It follows a known pattern of response to the suspected study procedure.

REMOTE (must have first two)

In general, this category is applicable to an AE that meets the following criteria:

1. It does not follow a reasonable temporal sequence of the study procedures.
2. It may readily have been produced by the subject's clinical state, environmental or toxic factors, or other modes of therapy administered to the subject.
3. It does not follow a known pattern of response to the suspected study procedure.
4. It does not reappear or worsen when the study procedure continues.

UNRELATED

This category is applicable to those AEs which are judged to be clearly and incontrovertibly due only to extraneous causes (disease, environment etc.) and do not meet the criteria for study procedures relationship listed under remote, possible or probable.

8.6 Reporting Requirements

8.6.1 Serious Adverse Event Reporting Requirements

Any adverse event (AE) that is serious, occurring during the course of the study, irrespective of the assessments performed on the subject, must be reported to the sponsor within one working day of its occurrence allowing expedited reporting.

The study will adhere to the full requirements of ICH Guidelines for Clinical Safety Data Management, Definitions and Standards for Expedited Reporting, Topic E2 will be adhered to. Complete information can be found in [Appendix xxx].

8.6.2 Non-Serious Adverse Event Reporting Requirements

As this is a non-therapeutic study, all AEs reported will be retained at the Investigator site as source notes.

Supplementary Table S1 – Malignancy types in Treated and Non-Treated groups

Cancer Type	Non-Treated Group (*n* (%))	Treated Group (*n* (%))
**Bone and Soft Tissue Malignancies**	**1 (5.0)**	**1 (1.2)**
**Gastrointestinal**	**5 (25.0)**	**5 (6.02)**
**Haematological**	**0 (0.0)**	**13 (15.66)**
**liver**	**5 (25.0)**	**1 (1.2)**
**Lung**	**0 (0.0)**	**50 (60.24)**
**Neuroendocrine Tumors (NET)**	**9 (45.0)**	**0 (0.0)**
**Neurological**	**0 (0.0)**	**1 (1.2)**
**oropharyngeal**	**0 (0.0)**	**12 (14.46)**

Supplementary Table S2. Chemotherapy Regimens

Control	Treated
Docetaxel<br>5-Fluorouracil<br>Gemcitabine<br>Cytarabine<br>Rituximab<br>Methotrexate<br>Vinorelbin<br>Epirubicine Hydrochloride<br>Ifosfamide<br>Dexrazoxane<br>Cyclophosphamide<br>Oxaliplatin	Cisplatin

Supplementary Table S3. 22 Biomarkers used prior to stage gate analysis

BIOMARKER LAB PARAMETER	N	CATEGORY	UNIT
ALBUMIN (URINE)	171	STANDARD	MG/L
ALPHA-1 MICROGLOBULIN	53	BIOMARKER	MG/L
ALPHA GLUTATHIONE S-TRANSFERASE	158	BIOMARKER	UG/L
CLUSTERIN	158	BIOMARKER	UG/L
CONNECTIVE TISSUE GROWTH FACTOR	53	BIOMARKER	UG/L
CREATININE (URINE)	171	STANDARD	UMOL/L
HUMAN COLLECTING DUCT ANTIGEN	53	BIOMARKER	U/L
INTERLEUKIN 18	53	BIOMARKER	NG/L
KIDNEY INJURY MOLECULE-1	158	BIOMARKER	UG/L
LIPOCALIN-2	53	BIOMARKER	UG/L
LIVER-FATTY-ACID-BINDING PROTEIN	53	BIOMARKER	UG/L
MONOCYTE CHEMOTACTIC PROTEIN 1	158	BIOMARKER	NG/L
OSTEOPONTIN	158	BIOMARKER	UG/L
PI GLUTATHIONE S-TRANSFERASE	53	BIOMARKER	UG/L
RETINOL BINDING PROTEIN 4	53	BIOMARKER	UG/L
TISSUE INHIBITOR OF METALLOPROTEINASE-1	53	BIOMARKER	UG/L
TOTAL PROTEINS (URINE)	172	STANDARD	MG/L
TREFOIL FACTOR 3	53	BIOMARKER	UG/L
UREA (URINE)	171	STANDARD	MMOL/L
URINARY COLLAGEN IV	53	BIOMARKER	UG/L
URINE CYSTATIN C	158	BIOMARKER	UG/L
URINE NEUTROPHIL GELATINASE-ASSOCIATED LIPOCALIN	158	BIOMARKER	NG/L

Supplementary Table S4. Biomarkers inclusion and exclusion reasons from the stage gate analysis

Reason for Biomarker exclusion or Inclusion	Biomarkers
Included due to clinical validation	Urine albumin/creatinine ration, Urine total protein, Serum cystatin C
Included due to pre-clinical validation	Kidney Injury Molecule-1 Clusterin, Cystatin C, Neutrophil gelatinase-associated lipocalin
Included due to excellent stage gate analysis AUC performance	α- Glutathione s-transferase, Osteopontin
Excluded due to poor stage gate analysis AUC performance	Trefoil Factor

Excluded due to absence of pre-clinical validation	Aquaporin, Calbindin, Collagen Iv, Connective Tissue Growth Factor, Human Collecting Duct Antigen, Interleukin 18, Liver-Fatty-Acid-Binding Protein, Monocyte Chemotactic Protein 1, Pi Glutathione S-Transferase, Tissue Inhibitor of Metalloproteinase-1

Supplementary Table S5. Assays formats for biomarkers chosen at stage gate analysis and biomarker main biological significance.

Selected Biomarker*	Main Biological Significance	Assay Format
Alpha GST	Proximal tubular necrosis marker	Single assay ELISA
Clusterin	Cell adhesion	Multiplex (Luminex)
Cystatin C	Proximal tubule reabsorbtion function	Multiplex (Luminex)
Cystatin C (serum)	Glomerular filtration rate	Single assay ELISA
KIM-1	Renal tubular inflammatory signaling molecule, regeneration	Multiplex (Luminex)
Microalbumin§	Glomerular and proximal tubular function	Single assay ELISA
NGAL	Renal tubular inflammatory signaling molecule	Multiplex (Luminex)
Osteopontin	Distal tubular injury marker	Multiplex (Luminex)

Supplementary Table S6. Assays formats for novel biomarkers

Biomarker	method	Provider, order number
Alpha GST	ELISA	Argutus Medical, Human Alpha GST EIA<br>*now*: Teco Medical Human Alpha GST EIA, # TE1056
Clusterin	Sandwich-Immunoassay	Rules Based Medicine (now Myriad RBM) Assays
Cystatin C	Sandwich-Immunoassay	Rules Based Medicine (now Myriad RBM) Assays
KIM-1	Sandwich-Immunoassay	Rules Based Medicine (now Myriad RBM) Assays
NGAL	Sandwich-Immunoassay	Rules Based Medicine (now Myriad RBM) Assays

Supplementary Table S7. Demographics and Subject Characteristics at Baseline

	Treated group (N=105)	Non-treated Group (N=20)
Age (years)		
Mean (SD)	59.5 (10.6)	63.4 (10.7)
Sex [n (%)]		
Race [n (%)]		
Caucasian/White	100 (95.2)	19 (95.0)
Black	1 (1.0)	0
Asian	1 (1.0)	0
Other	3 (2.9)	1 (5.0)
BMI (kg/m^2)		
Mean (SD)	25.37 (4.50)	26.14 (4.76)
Diabetes Mellitus		
Number (%)	12 (11.4)	6 (30)
Baseline Kidney Markers		
Serum Creatinine (mg/dL)(SD)	0.83 (0.24)	0.91 (0.35)
Blood Urea Nitrogen (mg/dL)(SD)	23.67 (10.17)	36.35 (22.25)
eGFR (ml/min)(SD)	101.07 (29.35)	79.83 (22.93)

Supplementary Figure 1. Median time to peak based per AKI group classification.

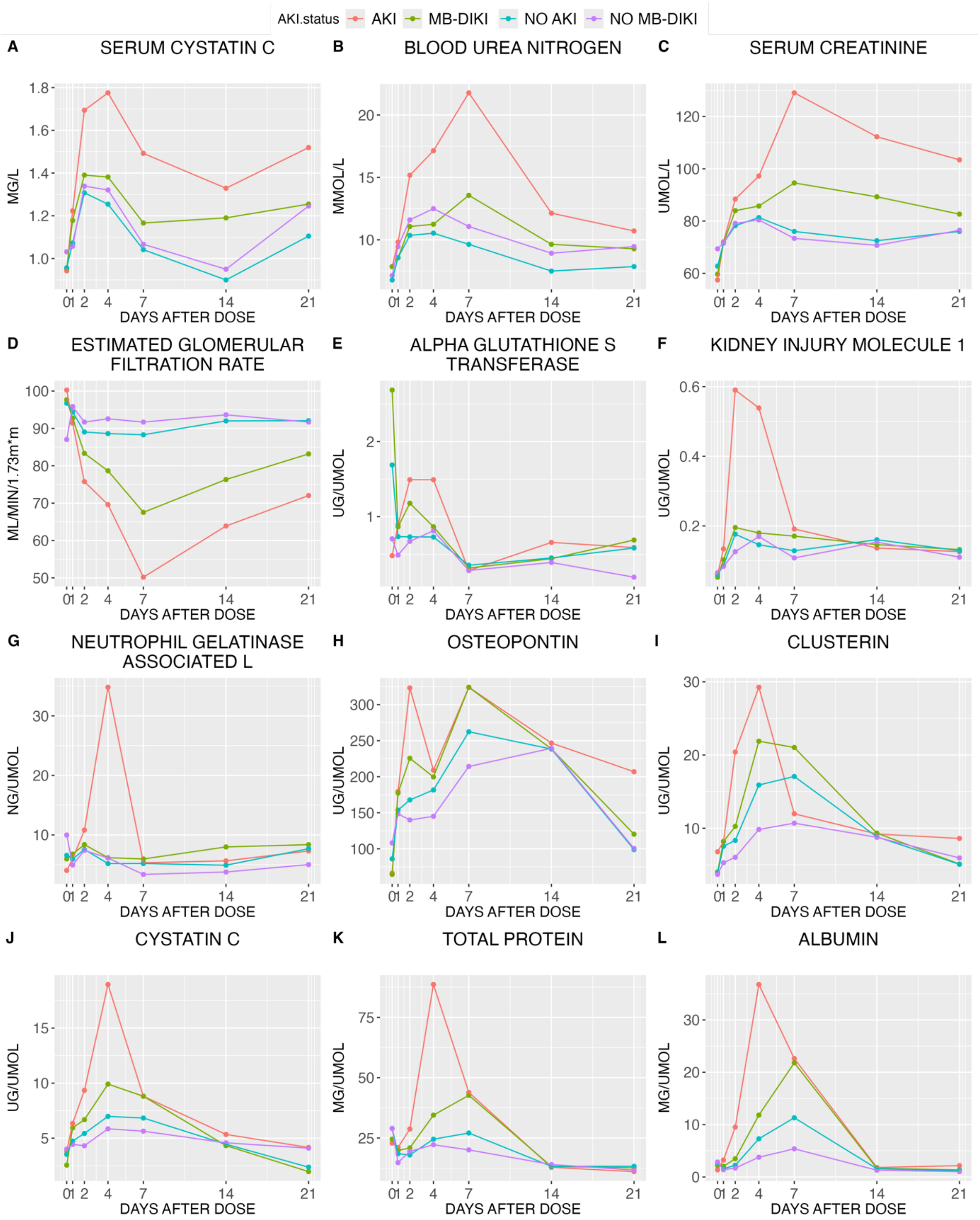

STROBE Checklist

STROBE Statement—checklist of items that should be included in reports of observational studies

	Item No.	Recommendation	Page No.	Relevant text from manuscript
**Title and abstract**	1	(*a*) Indicate the study's design with a commonly used term in the title or the abstract		
		(*b*) Provide in the abstract an informative and balanced summary of what was done and what was found	2	Methods section of abstract
**Introduction**				
Background/rationale	2	Explain the scientific background and rationale for the investigation being reported	3-4	Introduction section
Objectives	3	State specific objectives, including any prespecified hypotheses	4	Paragraph 5 of Introduction
**Methods**				
Study design	4	Present key elements of study design early in the paper	5	Paragraph 1 of Methods
Setting	5	Describe the setting, locations, and relevant dates, including periods of recruitment, exposure, follow-up, and data collection	5-6	Paragraph 1, Inclusion/exclusion criteria, and Sample collection
Participants	6	(*a*) *Cohort study*—Give the eligibility criteria, and the sources and methods of selection of participants. Describe methods of follow-up<br>*Case-control study*—Give the eligibility criteria, and the sources and methods of case ascertainment and control selection. Give the rationale for the choice of cases and controls<br>*Cross-sectional study*—Give the eligibility criteria, and the sources and methods of selection of participants	6	Inclusion/exclusion criteria, and Sample collection
		(*b*) *Cohort study*—For matched studies, give matching criteria and number of exposed and unexposed<br>*Case-control study*—For matched studies, give matching criteria and the number of controls per case		
Variables	7	Clearly define all outcomes, exposures, predictors, potential confounders, and effect modifiers. Give diagnostic criteria, if applicable	5-10	Throughout Methods subsections
Data sources/ measurement	8*	For each variable of interest, give sources of data and details of methods of assessment (measurement). Describe comparability of assessment methods if there is more than one group	*5-10*	Throughout Methods subsections
Bias	9	Describe any efforts to address potential sources of bias	9-10	Methods section – statistical analysis
Study size	10	Explain how the study size was arrived at	9	Second Paragraph of "statistical Analyis" section in methods

Quantitative variables	11	Explain how quantitative variables were handled in the analyses. If applicable, describe which groupings were chosen and why	9-10	statistical Analyis section in methods
Statistical methods	12	(*a*) Describe all statistical methods, including those used to control for confounding	9-10	statistical Analyis section in methods
		(*b*) Describe any methods used to examine subgroups and interactions	9-10	statistical Analyis section in methods
		(*c*) Explain how missing data were addressed	9	First paragraph of statistical analysis in methods
		(*d*) *Cohort study*—If applicable, explain how loss to follow-up was addressed<br>*Case-control study*—If applicable, explain how matching of cases and controls was addressed<br>*Cross-sectional study*—If applicable, describe analytical methods taking account of sampling strategy	9	First paragraph of statistical analysis in methods
		(*e*) Describe any sensitivity analyses	9-10	statistical Analyis section in methods
**Results**				
Participants	13*	(a) Report numbers of individuals at each stage of study—eg numbers potentially eligible, examined for eligibility, confirmed eligible, included in the study, completing follow-up, and analysed	11	Results: Characteristics of unadjudicated, Stage 1 adjudicated, and control groups Paragraph 1
		(b) Give reasons for non-participation at each stage	NA	NA
		(c) Consider use of a flow diagram	NA	NA
Descriptive data	14*	(a) Give characteristics of study participants (eg demographic, clinical, social) and information on exposures and potential confounders	11	Results: Characteristics of unadjudicated, Stage 1 adjudicated, and control groups section
		(b) Indicate number of participants with missing data for each variable of interest	NA	NA
		© *Cohort study*—Summarise follow-up time (eg, average and total amount)	NA	NA
Outcome data	15*	*Cohort study*—Report numbers of outcome events or summary measures over time	*11-14*	*Results*
		Case-control study—Report numbers in each exposure category, or summary measures of exposure		
		Cross-sectional study—Report numbers of outcome events or summary measures		
Main results	16	(*a*) Give unadjusted estimates and, if applicable, confounder-adjusted estimates and their precision (eg, 95% confidence interval). Make clear which confounders were adjusted for and why they were included	*11-14*	*Results*
		(*b*) Report category boundaries when continuous variables were categorized	*11-14*	*Results*

		(*c*) If relevant, consider translating estimates of relative risk into absolute risk for a meaningful time period		
Other analyses	17	Report other analyses done—eg analyses of subgroups and interactions, and sensitivity analyses		
**Discussion**				
Key results	18	Summarise key results with reference to study objectives	14	Discussion
Limitations	19	Discuss limitations of the study, taking into account sources of potential bias or imprecision. Discuss both direction and magnitude of any potential bias	14-16	Discussion
Interpretation	20	Give a cautious overall interpretation of results considering objectives, limitations, multiplicity of analyses, results from similar studies, and other relevant evidence	14-16	Discussion
Generalisability	21	Discuss the generalisability (external validity) of the study results	14-16	Discussion
**Other information**				
Funding	22	Give the source of funding and the role of the funders for the present study and, if applicable, for the original study on which the present article is based	16	Disclosures

*Give information separately for cases and controls in case-control studies and, if applicable, for exposed and unexposed groups in cohort and cross-sectional studies.

Note: An Explanation and Elaboration article discusses each checklist item and gives methodological background and published examples of transparent reporting. The STROBE checklist is best used in conjunction with this article (freely available on the Web sites of PLoS Medicine at http://www.plosmedicine.org/, Annals of Internal Medicine at http://www.annals.org/, and Epidemiology at http://www.epidem.com/). Information on the STROBE Initiative is available at www.strobe-statement.org.
