# Supplementary material for: Corrigendum to “Early and Sensitive Detection of Cisplatin-Induced Kidney Injury Using Novel Biomarkers” [Kidney International Reports Volume 10, Issue 4, April 2025, Pages 1175-1187]
Source: Kidney Int Rep. 2025 Dec 9;11(2):103723. doi: 10.1016/j.ekir.2025.103723 (PMC12804094; doi:10.1016/j.ekir.2025.103723)
Supplement: Supplementary Material [file mmc1.pdf]

IMI SAFE-T

Clinical exploratory PoT studies and clinical biomarker assay validation

Protocol

An exploratory, prospective, longitudinal, case control study to assess novel biomarkers of nephrotoxicity in patients receiving high dose cisplatin chemotherapy.

Author(s):	Dr Volker Schmitz, James Matcham, Dr Stefan Sultana
Document type:	Amendment 2
Document status:	Final
Document date:	02 April 2012

Property of IMI SAFE-T
Confidential
May not be used, divulged, published or otherwise disclosed
without the consent of IMI SAFE-T

Document history

Document type	Date
Final protocol	14 Apr 2011
Amendment 1	13 Jan 2012
Amendment 2	02 Apr 2012

Signatures

Authors:

Name

Principal Investigator	Dr. Volker Schmitz	date

Name

Study Biostatistician	James Matcham	date

Name

Study Clinician	Dr Stefan Sultana	date

1. INTRODUCTION

1.1 Background and Rationale

This study is part of a consortium-based clinical programme the aim of which is to develop and qualify novel safety markers of kidney, liver and vascular injury. The SAFE-T consortium is part of the Innovative Medicines Initiative and represents a collaboration between academic institutions, pharmaceutical companies and related small business enterprises, and the European Medicines Agency. The primary aim of this consortium is to develop novel safety markers that can be used both in drug development programmes to monitor organ safety as well as in hospital practice to identify patients at risk of organ injury or detect this injury at an earlier stage and therefore improve the prognosis by prompt intervention and treatment.

Blood urea nitrogen (BUN), serum creatinine and urinary protein (either by dipstick urinalysis or measuring urinary protein/ creatinine ratio) are currently used to monitor renal safety and to detect acute renal injury. Whilst these renal markers are highly specific, at least 50% of renal function may be lost before these markers are able to detect any injury. This can be seen in the case of live donors in renal transplantation where the donor's BUN and creatinine do not usually show any significant change from pre-operative levels. Furthermore, these markers do not usually change until 3 to 5 days after the injury. There is therefore a need for more sensitive markers to detect renal injury at a milder and earlier stage.

Cisplatin is a widely used and potent chemotherapy drug administered alone or in combination for the treatment of a number of cancers such as testicular, head and neck, ovarian, cervical and non-small cell lung carcinoma, amongst other [Pabla and Dong, 2008]. Its use is limited by adverse effects, notably nephrotoxicity. This occurs in about one-third of patient undergoing high dose cisplatin treatment. Clinically, nephrotoxicity is often seen within about 10 days of cisplatin administration and is manifested as lower glomerular filtration rate, higher serum creatinine, and reduced serum magnesium and potassium levels. It causes proximal and distal convoluted tubular damage by entering tubular cells and activating apoptotic pathways that result in cell death. It also induces the production of TNF, triggering a robust inflammatory response and further contributing to tubular cell injury and death. Cisplatin may also induce injury in the renal vasculature, leading to ischemic tubular cell death and decreased glomerular filtration rate (GFR). Together, these pathological events culminate in acute renal failure in a proportion of patients [Pabla and Dong, 2008].

1.2 Study Aim

The primary focus of the study is to assess novel markers of renal safety. Stored samples from this study will also be available for future research in renal safety markers as well as those of other organ systems.

The study will enrol cancer patients who are scheduled to receive high dose cisplatin as monotherapy or as part of a combination chemotherapy regimen. The cisplatin treatment will be administered as part of the standard of care management of these patients' cancer; there will not be any change to patients' cancer treatment as a result of participating in this study. Control patients and healthy volunteers will be recruited for comparison purposes. Blood and urine samples will be collected at baseline and at various time-points after the first administration of cisplatin to assess the time-course

of any changes in the various novel renal injury biomarkers and compare these with BUN and serum creatinine changes.

2. STUDY OBJECTIVES AND ENDPOINTS

2.1 Objectives

- To collect blood and urine samples in cisplatin-treated patients and control subjects.
- To characterise the within- and between-subject baseline variability of novel biomarkers relative to BUN/ serum creatinine in these populations.
- To compare the patterns of novel biomarker changes relative to BUN/ serum creatinine following cisplatin treatment. These data will be used to:
    - select candidate biomarkers to progress to the confirmatory stage of biomarker qualification and establish cut-off values for these biomarkers.
    - determine the optimum method of expressing (corrected vs. uncorrected) each novel urinary marker relative to urinary creatinine for subsequent studies.
    - characterise the time course of biomarker changes to optimise the study design and sample collection time-points of confirmatory studies.

2.2 Endpoints

- For each novel serum and urinary biomarker, as well as for BUN and serum creatinine, the following will be determined:
    - Maximum change from baseline.
    - Time to maximum change from baseline.
    - Once cut-off values are determined, time to abnormal change.
    - Time to return to baseline (or to return to normal range).
    - Mean change over period of observation (AUEC analysis)

- Novel urinary biomarkers will be assessed in the following ways in the various analyses of interest:
    - corrected for urinary creatinine concentration, and
    - uncorrected biomarker concentration.

3. STUDY DESIGN

This is a prospective, non-randomised, longitudinal, case control study. Patients with cancer who are scheduled to receive high dose cisplatin as part of their standard of care treatment (e.g. non-small cell lung cancer, head & neck cancer) will be enrolled in this study. Control patients with similar cancers who have not had recent major surgery or treatment with nephrotoxic drugs will be enrolled as a control patient population. A cohort of healthy subjects will be enrolled as a separate healthy control population.

Subjects will have a screening assessment where their diagnosis and eligibility for enrolment is confirmed. The study will be explained to them and subjects willing to participate will be asked to give informed consent. Unless documented by clinical

chemistry laboratory results in the previous month, subjects will have a blood sample taken for BUN and serum creatinine to document baseline renal function and confirm they meet the criteria for the study. Eligible patients scheduled to receive cisplatin will have blood and urine samples taken pre-dose on the day of first cisplatin administration and at intervals over the next 3 weeks. Control subjects (cancer patients and healthy volunteers) will attend the clinic to give blood and urine samples at two visits that are 2 days (+/- 1 day) apart.

Enrolled subjects will have blood and urine samples collected for novel biomarkers at the following time-points:

Cisplatin-treated group:

- Pre-dose on day of 1st cycle cisplatin chemotherapy administration (baseline)
- Prior to leaving the clinic following cisplatin administration (or at approximately 8 - 12 hours post-dose for in-patients)
- 24 hours post-dose
- 2 days post-dose
- 4 days (+/- 1 day) post-dose
- 7 days (+/- 1 day) post-dose
- 14 days (+/- 3 days) post-dose
- 21 days (+/- 7 days) post-dose

Control groups (cancer patients and healthy volunteers):

- Visit 1 (within 3 weeks of Screening: this may be the Screening visit after subjects have given their informed consent. In this case, for those subjects who are subsequently found not to qualify for inclusion after lab results are available, stored samples will be discarded and not included in any analysis. Any subjects not included after Screening will not count towards the number of enrolled subjects for the relevant cohort even if samples are taken and later discarded.)
- Visit 2 (2 days [+/- 1 day] after Visit 1)

Note that apart from samples taken in the cisplatin-treated patients in the 12 hour period after cisplatin infusion, all blood and urine samples will be collected in the morning. All subjects will be asked to attend the clinic within a 2-hour window (chosen after consultation between the Investigator and the subject) at each visit as far as possible.

Table 1: Schedule of Activities for cisplatin-treated group of patients

**Cisplatin-treated patient group**	Screening	Pre-dose	<12 hrs	24 hrs	2 days	4 days	7 days	14 days	21 days
Informed consent	x								
Blood sample (5 mL) for clinical chemistry	x	x	x	x	x	x	x	x	x
Urine sample (10 mL) for urinalysis	x	x	x	x	x	x	x	x	x
Check subject eligibility	x								
Review adverse events and concomitant medications	x	x	x	x	x	x	x	x	x
Enrol subject		x							
Blood sample (10 mL) for serum[1]		x	x	x	x	x	x	x	x
Blood sample (10 mL) for plasma[2]		x					x		
Urine sample (40 mL)[1]		x	x	x	x	x	x	x	x
Ensure patient has oncology follow-up visit									x

[1]To analyse for DIKI biomarkers as listed in Table 3.
[2]A plasma sample will be stored for future use for novel renal and other organ safety biomarkers.

Table 2: Schedule of Activities for patient controls

**Patient control group n=20**	Screening [2]	Visit 1[2]	Visit 2 (2 days post V1)
Informed consent	x		
Blood sample (5 mL) for clinical chemistry	x	x	X
Urine sample (10 mL) for urinalysis	x	x	X
Check subject eligibility	x		
Review adverse events and concomitant medications	x	x	X
Enrol subject		x	
Blood sample (10 mL) for serum[1]		x	X
Blood sample (10 mL EDTA) for plasma		x	X
Urine sample (40 mL) [1]		x	X

[1]To analyse for DIKI biomarkers as listed in Table 3.
[2]These visits may be combined if subject gives informed consent

Table 3: Schedule of Activities for healthy control group

**Healthy control group n=20**	Screening [2]	Visit 1[2]	Visit 2 (2 days post V1)	Visit 3 (14 to 28 d post V1)
Informed consent	x			
Blood sample (5 mL) for clinical chemistry	x	x	X	x
Urine sample (10 mL) for urinalysis	x	x	X	x
Check subject eligibility	x			
Review adverse events and concomitant medications	x	x	X	x
Enrol subject		x		
Blood sample (10 mL) for serum[1]		x	X	x
Blood sample (10 mL EDTA) for plasma		x	X	x
Urine sample (40 mL) [1]		x	X	x

[1]To analyse for DIKI biomarkers as listed in Table 3.
[2]These visits may be combined if subject gives informed consent

This is an observational study. All patients in this study will receive treatment as per standard of care for their conditions. Patients in this study that develop BUN or serum creatinine changes following cisplatin treatment that qualify as acute kidney injury will be managed according to standard hospital treatment protocols.

4. SUBJECT SELECTION

4.1 Inclusion Criteria

All subjects

- Males and females ≥18 years of age.

Cisplatin patients

- Patients with documented cancer (e.g. head & neck or similar) who are scheduled to receive 1st cycle high dose cisplatin therapy (≥65 mg/ m^2/ cycle).

Control subjects

- Patients with documented cancer (similar to patients due to be treated with cisplatin) who have received or are scheduled to receive non-nephrotoxic treatment modalities.
- A further group of healthy control subject will be enrolled for comparison.

4.2 Exclusion Criteria

All subjects

- Chronic kidney disease defined by microalbuminuria (>30 mcg/ mg urinary creatinine) or eGFR <60 mL/min/1.73m^2.
- Regular co-administration of any of the following within 7 days prior to screening until the last sample collection time-point.
  - creatine supplements
  - drugs known to alter tubular secretion of creatinine (e.g. trimethoprim, cimetidine).
  - Non-steroidal anti-inflammatory drugs (ibuprofen, diclofenac, naproxen; occasional use allowed).
- Major surgery from 1 month prior to screening until the last sample collection time-point.
- *NOTE*: patients needing to take diuretic, ACEi or ARB drugs may be enrolled and continue on these therapies but these patients will be analysed separately.
- Inability to comprehend, or unwillingness to follow, the study requirements including attendance at out-patient clinic visits and participation in laboratory testing as called for by the protocol.

4.3 Life Style Guidelines

Subjects should avoid strenuous exercise during the 24 hours preceding each biosample collection. Walking, moderate cycling and similar activities are allowed.

5. STUDY TREATMENTS

5.1 Allocation to Treatment

This is an observational study. All patients in this study will receive the standard of care protocol of treatment for their specific malignancy. Healthy subjects will not receive any treatment. Subjects will be enrolled into the study based on whether they are scheduled to receive high dose cisplatin therapy (cisplatin group), non-nephrotoxic therapy (control cancer group) or no treatment (healthy control group).

5.2 Drug Supplies

Standard of care treatment will be provided by the hospital or other institutions where the patients are being treated.

5.3 Concomitant Medication

If any patients require treatment with drugs that are known to be nephrotoxic (e.g. aminoglycoside antibiotics, chronic use NSAIDs) or that interfere with creatinine secretion (e.g. trimethoprim, cimetidine), they will be withdrawn from the study. Any samples collected before they receive any such medications will be included in any pooled analyses of data.

5.4 Rescue/Salvage Therapy

Patients who develop signs or lab test changes that qualify as acute kidney injury will be managed according to the standard protocols of treatment for that institution. This includes both active management and longer-term follow-up of these patients.

The patients' cancer therapy (based on response/non-response to cisplatin for example) will be managed according to that institutions treatment policy for each specific cancer.

6. STUDY PROCEDURES

6.1 Screening

Subjects eligible for the study will have the nature and purpose of the study explained to them by the investigator. They will be provided a written copy of the informed document for the study and given sufficient time to consider the study's implications (e.g. sample collection schedule) before deciding to participate.

The subjects' medical history, prior and concurrent medications, and demographics will be documented. A full physical examination will be done, including blood pressure, body weight and height. A blood sample for BUN and serum creatinine will be taken unless documented by results are available in the previous month. An urine sample will be obtained for routine urinalysis. Eligibility will be determined by checking the inclusion/exclusion criteria.

6.2 Study Visits

Cisplatin treatment group

The cisplatin treatment group of subjects will have blood and urine samples collected as described below. Details of samples collected are given in Section 7.

- Pre-dose blood and urine sample collection before 1st dose of cisplatin chemotherapy.

Patients will then receive the cisplatin infusion as per the standard treatment protocol for their institution. The date and time of starting cisplatin infusion will be recorded in the Case Record Form.

- Blood and urine sample collection within first 12 hours post-1st dose of cisplatin chemotherapy.

Patients who attend for cisplatin treatment on an outpatient basis will have the post-dose set of blood and urine samples collected just before going home. The time of this sample collection will be recorded in the Case Record Form.

For patients who are receiving cisplatin as in-patients, this first post-dose sample should be taken approximately 8 to 12 hours after the start of the cisplatin infusion.

For the 3 weeks following the first cisplatin infusion, patients will attend the clinic on the days described below. Samples should be collected when they arrive in the clinic. If a patient is unable to produce a urine specimen on arrival at the clinic, he/she should be given some fluids to drink in order to produce the sample. As far as possible, clinic visits should be in the morning and should be arranged such that they are within a 2 hour window that is convenient for the patient and clinic staff.

Blood and urine sample collections at:
- 24 hours post-dose.
- 2 days (i.e. 48 hours) post-dose.

Patients should make every effort to attend to give samples at both 24 and 48 hours post-dose. If patients are not able to attend for either time-point, they should be withdrawn from the study and no further samples collected.

- 4 days (+/- 1 day) post-dose.
- 7 days (+/- 1 day) post-dose.
- 14 days (+/- 3 days) post-dose.
- 21 days (+/- 7days) post-dose.

Patients who miss an appointment to give blood and urine samples should be contacted to see if the visit can be rescheduled. If visits cannot be rescheduled and a patient misses more than 2 visits, they should be withdrawn from the study and no further samples collected.

Control groups

Control subjects (non-cisplatin treated cancer patients, healthy volunteers) will have blood and urine samples collected as described below. Note that for cancer control group patients who are receiving inpatient treatment, morning blood and urine will be collected in the hospital or clinic they are being treated in. Details of samples collected are given in Section 7.

Blood samples and a urine sample will be collected when subjects arrive in the clinic. If a subject is unable to produce a urine specimen on arrival at the clinic, he/she should be given some fluids to drink in order to produce the sample. As far as possible, clinic visits should be in the morning and should be arranged such that they are within a 2 hour window that is convenient for the patient and clinic staff.

Samples should be provided on:

- Visit 1: this should be within 3 weeks of the Screening assessment (see previous regarding combining Screening and Visit 1).
- Visit 2: this should 2 days (+/- 1 day) after Visit 1.

6.3 Follow-up Visit

Since this is an observational study, there will be no follow up visit. Cancer patients (both cisplatin-treated group and control patients) will be followed up and treated according to the standard of care protocol of their hospital or institution. The investigator should check that patients have an appointment for their next cancer treatment or clinic visit as appropriate.

6.4 Subject Withdrawal

Subjects can withdraw from the study at any time. There will be no study follow up visit and cancer patients will revert to the usual hospital or clinic visit schedule as per their standard of care.

7. SAMPLE COLLECTION & HANDLING

10 mL blood (serum) and 40 mL urine samples will be collected at each visit for the following kidney biomarkers:

Table 3: Novel biomarkers to be measured in this study

**Type of biomarker**	**Biomarker name***	**Main significance**
Functional biomarkers	Microalbumin/Albumin	Marker of impaired proximal tubular reabsorption
	α-1 microglobulin	Marker of impaired proximal tubular reabsorption and indirectly of glomerular injury
	Cystatin C	Evaluation of glomerular filtration rate (serum)<br>Marker of impaired proximal tubular reabsorption and indirectly of glomerular injury(urine)
	Urinary creatinine	Marker of impaired proximal tubular reabsorption and indirectly of glomerular injury(urine)
	Retinol Binding Protein-4 (RBP-4)	Marker of impaired proximal tubular reabsorption
Tissue injury leakage markers	N-acetyl-β-D-glucosaminidase (NAG)	Marker of proximal tubular injury
	Glutathione-S-transferase-α (GST-α)	Marker of proximal tubular injury
	Glutathione-S-transferase-π (GST-π)	Marker of distal tubular injury
	Liver-type fatty acid binding protein (L-FABP)	Marker of proximal tubular injury
	Collagen IV	Marker of glomerular injury
	Podocin	Marker of glomerular injury
	Nephrin	Marker of glomerular injury
	Aquaporin-2	Marker of collecting duct injury
	Calbindin D28	Marker of injury to distal regions of nephron and collecting ducts
Tissue injury response	Kidney injury molecule-1	Marker of proximal tubular

Type of biomarker	Biomarker name*	Main significance
markers	(KIM-1)	injury/regeneration
	Clusterin	Marker of tubular injury/regeneration (no apparent specific nephronal localization)
	Neutrophil gelatinase associated lipocalin (NGAL)	Marker of tubular (mainly proximal) injury
	Trefoil Factor 3 (TFF3)	Marker of proximal tubular injury
	Osteopontin	Marker of injury to distal regions of nephron
	Tissue inhibitor of metalloproteinase-1 (TIMP-1)	Marker of interstitial fibrosis and tubular injury
	Connective Tissue Growth Factor (CTGF)	Marker of interstitial fibrosis
	Interleukin-18 (IL-18)	Marker of inflammation
	Monocyte chemoattractant protein-1 (MCP-1)	Marker of inflammation

* All biomarkers will be assessed in urine and Cystatin C and NGAL will additionally be assessed in plasma/serum.

A 10 mL EDTA blood sample will be taken at the baseline and Day 7 visits only. The plasma obtained from these samples will be stored for future use for novel renal and other organ safety biomarkers.

Additional the following parameters will be assessed in the 5 ml blood plus 10 ml urine for routine diagnostics.

Blood Chemistry	Total protein, Albumin, Creatinine, Urea<br>Sodium, Chloride, Calcium, Phosphate, Potassium, Magnesium
Urinalysis	U-Kreat; U-Urea; Na, K, U-Protein, U-Albumin (Midstream)

Table 4: Type of Samples and Approximate Amount of Blood and Urine to be collected from Each Subject

Test	Sample volume (mL)	Number of visits sampled		Total volume (mL)	
		Cisplatin group	Control groups	Cisplatin group	Control groups
Routine laboratory test	5	9	3	45	15
DIKI serum sample	10	8	2	80	20
DIKI ETDA blood	10	2	2	20	20
**Total blood volume**	**-**	**-**	**-**	**145**	**55**
Routine urinalysis	10	9	3	90	30
DIKI urine sample	40	8	2	320	80
**Total urine volume**	**-**	**-**	**-**	**410**	**110**

Total Blood Loss

Approximately 145 mL whole blood will be drawn in patients receiving cisplatin treatment and approximately 55 ml whole blood will be drawn in control subjects (healthy volunteers and cancer control patients).

Sample Handling Procedures

Details on blood and urine sample handling and storage at the site as well as shipment conditions for frozen samples are detailed in a separate sample handling SOP.

8. ADVERSE EVENT REPORTING

8.1 Definition of Adverse Events

An Adverse Event (AE) is any untoward medical occurrence in a subject or clinical investigation subject administered a pharmaceutical product and which does not necessarily have to have a causal relationship with the intervention. An AE can therefore be any unfavourable and unintended sign (including an abnormal laboratory finding, for example), symptom, or disease temporally associated with the use of a pharmaceutical product, whether or not considered related to the study. Pre-existing conditions that worsen during a study are to be reported as AEs.

8.2 Reporting Period

All clinical adverse events (AEs) encountered during the clinical study will be recorded on AE source documents and retained at the Investigator site.

8.3 Serious Adverse Events

A serious adverse event (SAE) is any experience that suggests a significant hazard, contraindication, side effect or precaution. An SAE must fulfill at least one of the following criteria:

- is fatal (results in the outcome death)
- is life-threatening
- required in-patient hospitalization or prolongation of existing hospitalization
- results in persistent or significant disability/incapacity
- is a congenital anomaly/birth defect
- is medically significant or requires intervention to prevent one or other of the outcomes listed above

Serious Adverse Events unrelated to the study procedure and /or underlying conditions, must be collected and reported during the study and up to 15 days after the last visit.

8.4 Severity Assessment

All clinical adverse events (AEs) encountered during the clinical study will be recorded on AE source documents and retained at the Investigator site. Intensity of AEs will be graded on a four–point scale (mild, moderate, severe and life threatening). A description of scales can be found below.

- Mild discomfort noticed but no disruption of normal daily activity
- Moderate discomfort sufficient to reduce or affect daily activity
- Severe inability to work or perform normal daily activity
- Life Threatening represents an immediate threat to life

Relationship of the AE to the study procedure or prescribed medications should also be assessed. Details can be found below.

8.5 Causality Assessment

Relationship of the AE to the study procedure or prescribed medications should be assessed as probable, possible, remote or unlikely.

PROBABLE (must have first three)

This category applies to those AEs that are considered, with a high degree of certainty, to be related to the study procedures. An AE may be considered probable, if:

1. It follows a reasonable temporal sequence of the study procedures.
2. It cannot be reasonably explained by the known characteristics of the subject's clinical state, environmental or toxic factors, or other modes of therapy administered to the subject.
3. It disappears or decreases on cessation or reduction of the study procedures.
4. It follows a known pattern of response to the suspected study procedure.
5. It reappears upon rechallenge.

POSSIBLE (must have first two)

This category applies to those AEs in which the connection with the study procedures appears unlikely but cannot be ruled out with certainty. An AE may be considered possible if, or when:

1. It follows a reasonable temporal sequence of the study procedures.
2. It may have been produced by the subject's clinical state, environmental or toxic factors, or other modes of therapy administered to the subject.
3. It follows a known pattern of response to the suspected study procedure.

REMOTE (must have first two)

In general, this category is applicable to an AE that meets the following criteria:

1. It does not follow a reasonable temporal sequence of the study procedures.
2. It may readily have been produced by the subject's clinical state, environmental or toxic factors, or other modes of therapy administered to the subject.
3. It does not follow a known pattern of response to the suspected study procedure.
4. It does not reappear or worsen when the study procedure continues.

UNRELATED

This category is applicable to those AEs which are judged to be clearly and incontrovertibly due only to extraneous causes (disease, environment etc.) and do not meet the criteria for study procedures relationship listed under remote, possible or probable.

8.6 Reporting Requirements

8.6.1 Serious Adverse Event Reporting Requirements

Any adverse event (AE) that is serious, occurring during the course of the study, irrespective of the assessments performed on the subject, must be reported to the sponsor within one working day of its occurrence allowing expedited reporting.

The study will adhere to the full requirements of ICH Guidelines for Clinical Safety Data Management, Definitions and Standards for Expedited Reporting, Topic E2 will be adhered to. Complete information can be found in [Appendix xxx].

8.6.2 Non-Serious Adverse Event Reporting Requirements

As this is a non-therapeutic study, all AEs reported will be retained at the Investigator site as source notes.

9. DATA ANALYSIS/STATISTICAL METHODS

9.1 Sample Size

The maximum sample size for this multiple cohort observational study is 140 patient/subjects comprising;

- 100 patients in the cisplatin cohort to have a minimum of 20 subjects who show post-cisplatin BUN and serum creatinine changes that qualify as AKI.
- 20 patients in the patient control cohort.
- 20 subjects in the volunteer control cohort.

A sample size of 100 patients in the cisplatin cohort and 20 patients/subjects in each control cohort will enable a reliable assessment of the form of the statistical distribution for baseline values for each novel BM. In addition, a sample size of 20 cisplatin patients with AKI and 20 patients/subjects in each control cohort will enable an exploratory comparison between cohorts to detect patterns of difference in the BM profiles over time.

The sample size of the cisplatin-treated and the control cohorts may be adjusted during the course of the study based on interim analyses of novel biomarker patterns as well as the incidence of AKI as defined by BUN and serum creatinine changes in the cisplatin cohort.

9.2 Statistical Analysis

9.2.1 Analysis of Primary Endpoint

The full list of novel serum and urinary biomarkers (BM) included in the analysis are provided in Table 3 (Section 7).

The statistical distribution of the baseline BM values will be described graphically and by calculating the mean, median, standard deviation, inter-quartile range and range within each cohort and overall. The form of the relationships between each pair of BMs will also be investigated.

For each BM, the profile of values over time will be summarised and inspected to see if any patterns of change differ between the cisplatin cohort and the two control cohorts. BMs that show a different response between cohorts will be further investigated to characterise the form of the response and to evaluate if the response appears associated with concurrent changes in serum creatinine and BUN.

BMs will be assessed, one at a time, for their ability to predict DIKI as defined by the AKIN and RIFLE criteria[refs]. In addition, each patient in the cisplatin cohort will be reviewed by an adjudication panel to assess presence of DIKI using all of the available data. BMs will also be assessed for their ability to predict DIKI as defined by the adjudication panel. Exploratory ROC curves will be generated and estimates of sensitivity and specificity will be given. BMs will be ranked according to their estimated probability of having a sensitivity and specificity of greater than 80% for predicting DIKI.

Exploratory analyses may also be conducted to evaluate the potential for combining BMs to enhance prediction of DIKI.

9.2.2 Analysis of Secondary Endpoints

The secondary endpoints are

- BUN
- Serum Creatinine
- Incidence of DIKI (as defined by AKIN, RIFLE and the Adjudication Panel).

The baseline values for BUN and serum creatinine will be summarised graphically and by calculating the mean, median, standard deviation, interquartile range and range within each cohort and overall. The profile of values of BUN and serum creatinine over time will be summarised and inspected to see if any patterns of change differ between the cisplatin cohort and the two control cohorts.

The number and percentage of patients/subjects that meet the different criteria for DIKI will be calculated for each cohort.

9.3 Safety Analysis

The incidence of clinical DIKI events will be summarised by calculating the number and percentage of patients/subjects with each clinical DIKI event. Narratives of each event will also be given. Clinical DIKI events will be coded using the MEDDRA V.x dictionary before analysis.

9.4 Interim Analysis

An interim analysis of the biomarker endpoints will take place after the data from 30 cisplatin cohort patients are entered on to the database and are available for analysis.

10. DATA HANDLING AND RECORD KEEPING

Data for this study will be recorded via an Electronic Case Report Form.

This study will be conducted in accordance with the protocol and in accordance with:

- The declaration d'Helsinki of 1964.
- The international rules and regulations governing GCP (1996)
- The charter on ethical research in developing countries edited by the ANRS (May 2002)Subject Information and Consent

11. ETHICAL CONSIDERATIONS

11.1 Informed consent

The patients will be informed in a transparent and complete fashion, in layman's terms, of the objectives and constraints of the study, of the potential risks associated with the study, of the monitoring, of the patient's right to decide not to participate in the study and of the patient's right to withdraw from the study at any time in the future. All this information will be contained in the patient information sheet in the same document as the informed consent form. A written informed consent by the patients will be collected by the investigator, or a medical representative of this investigator, before recruitment into the study. A copy of the patient information sheet and of the signed informed consent form, signed by both the patient and the investigator, or the investigator's representative, will be given to the patient, the investigator keeping one of the original copy. At the end of the study, one copy of this information sheet and consent form will be placed in a sealed envelope including all the other signed informed consent forms of the patients recruited into the study, and this envelope will be archived by the sponsor.

11.2 Independent Ethics Committees(IEC)Institutional Review Board(IRB)

The protocol, informed consent and any accompanying material provided to the subject will be submitted by the Investigator to an IRBfor review. An approval letter or certificate (specifying the protocol number and title) from the IEC/IRB must be obtained before study initiation by the Investigator specifying the date on which the committee met and granted the approval. This applies whenever subsequent amendments/modifications are made to the protocol.

12. ACCESS TO STUDY DATA AND SOURCE DOCUMENTS

Patients' data collected during the study will be kept strickly confidential and anonymous. Only the medical and scientific investigators representative of the sponsor, involved in the conduct of the study, in addition to a representative person from the health authority will be allowed to access the patients' medical source data. However, they will only be allowed to access this data if it is to audit the study. The data collected during the study will be digitized for IT storage.

13. QUALITY CONTROL AND QUALITY ASSURANCE

In accordance with Good clinical practice (GCP), and in order to guarantee the quality of the study and protect the patients recruited in the study, the investigator of the study will commit to

- the regular monitoring and audit of the study by a representative of the coordinating methodology committee of the study. This representative will have access to the patient source document in order to validate the data collected in the Case Report Form (CRF) (at any time, the principal investigator or his representative will be able to be contacted for any question related to the protocol, a logistical issue related to the protocol, and practical

prosecution of the protocol, or about any advice on management issues following a specific event in the study.

- An eventual audit of the study by the sponsor, or with the sponsor's authorization, an audit conducted by relevant organisations
- An audit of the study by the health authorities.

A CRF will be assigned to each patient recruited in the study, with a study number and the code linking this study number to the identification of the patient.

13.1 Retention of the documents and data related to the study:

The investigators in the study will archive the documents related to the study, and will keep these documents available for any potential audit for at least 15 years.

Such documents related to the study are as follows:

- updated version of the protocols, appendices, with any amendments.
- CRF
- Informed consent signed by the patients and the confidential list of patients having taking part in the study, linking each patient to the study number and the patient's source hospital case notes.
- Correspondence related to the study

The source data file must be available for a period of 15 years.

14. REFERENCES

Pabla N. and Dong Z. (2008). Cisplatin nephrotoxicity: mechanisms and renoprotective strategies. *Kidney Int.* 73, 994-1007.

Appendix 1: Amendments

Amendment 1 (24 Jan 2012)

Reasons for Amendment

- To amend the exclusion criteria to allow the enrolment of subjects that take infrequent or occasional NSAID medication.
- To refine the exclusion criterion regarding the time-period around major surgery.

Amended text: Page 7; Section 4.2 Exclusion Criteria

Old text (deleted text in strikethrough)

- Co-administration of any of the following from 7 days prior to screening until the last sample collection time-point.
  - creatine supplements,
  - drugs known to alter tubular secretion of creatinine (e.g. trimethoprim, cimetidine).
- ~~Major surgery or co-administration of drugs known to be nephrotoxic (i.e. chronic use of NSAIDs, aminoglycosides) from 1 month prior to screening.~~

New Text (added text in bold)

- **Regular** co-administration of any of the following within 7 days prior to screening until the last sample collection time-point.
  - creatine supplements
  - drugs known to alter tubular secretion of creatinine (e.g. trimethoprim, cimetidine).
  - **Non-steroidal anti-inflammatory drugs (ibuprofen, diclofenac, naproxen; occasional use allowed).**
- **Major surgery from 1 month prior to screening until the last sample collection time-point.**

Amendment 2 (02 Apr 2012)

Reason for Amendment

- To reduce the minimum dose of cisplatin to be administered to ≥65mg/ m^2/ cycle. This will allow enrolment of other patient groups and improve recruitment into the study while still limiting the population to those who receive a bolus administration of high dose cisplatin that is associated with a higher incidence of AKI.

Amended text: Page 7; Section 4.1 Inclusion Criteria

Changed text (deleted text in strikethrough and replacement text in bold)

Cisplatin patients

- Patients with documented cancer (e.g. head & neck or similar) who are scheduled to receive 1st cycle high dose cisplatin therapy (~~≥75~~ **≥65** mg/ m^2/ cycle).
